# Prediction of *Mycobacterium tuberculosis* pyrazinamidase function based on structural stability, physicochemical and geometrical descriptors

**DOI:** 10.1371/journal.pone.0235643

**Published:** 2020-07-31

**Authors:** Rydberg Roman Supo-Escalante, Aldhair Médico, Eduardo Gushiken, Gustavo E. Olivos-Ramírez, Yaneth Quispe, Fiorella Torres, Melissa Zamudio, Ricardo Antiparra, L. Mario Amzel, Robert H. Gilman, Patricia Sheen, Mirko Zimic

**Affiliations:** 1 Laboratorio de Bioinformática, Biología Molecular y Desarrollos Tecnológicos, Facultad de Ciencias y Filosofía, Universidad Peruana Cayetano Heredia, Lima, Peru; 2 Department of Biophysics and Biophysical Chemistry, Johns Hopkins University, Baltimore, MD, United States of America; 3 International Health Department, Johns Hopkins School of Public Health, Baltimore, MD, United States of America; Weizmann Institute of Science, ISRAEL

## Abstract

**Background:**

Pyrazinamide is an important drug against the latent stage of tuberculosis and is used in both first- and second-line treatment regimens. Pyrazinamide-susceptibility test usually takes a week to have a diagnosis to guide initial therapy, implying a delay in receiving appropriate therapy. The continued increase in multi-drug resistant tuberculosis and the prevalence of pyrazinamide resistance in several countries makes the development of assays for prompt identification of resistance necessary. The main cause of pyrazinamide resistance is the impairment of pyrazinamidase function attributed to mutations in the promoter and/or *pncA* coding gene. However, not all *pncA* mutations necessarily affect the pyrazinamidase function.

**Objective:**

To develop a methodology to predict pyrazinamidase function from detected mutations in the *pncA* gene.

**Methods:**

We measured the catalytic constant (k_cat_), K_M_, enzymatic efficiency, and enzymatic activity of 35 recombinant mutated pyrazinamidase and the wild type (Protein Data Bank ID = 3pl1). From all the 3D modeled structures, we extracted several predictors based on three categories: structural stability (estimated by normal mode analysis and molecular dynamics), physicochemical, and geometrical characteristics. We used a stepwise Akaike’s information criterion forward multiple log-linear regression to model each kinetic parameter with each category of predictors. We also developed weighted models combining the three categories of predictive models for each kinetic parameter. We tested the robustness of the predictive ability of each model by 6-fold cross-validation against random models.

**Results:**

The stability, physicochemical, and geometrical descriptors explained most of the variability (R^2^) of the kinetic parameters. Our models are best suited to predict k_cat_, efficiency, and activity based on the root-mean-square error of prediction of the 6-fold cross-validation.

**Conclusions:**

This study shows a quick approach to predict the pyrazinamidase function only from the *pncA* sequence when point mutations are present. This can be an important tool to detect pyrazinamide resistance.

## Introduction

Tuberculosis (TB) is a major infectious disease caused by *Mycobacterium tuberculosis* (MTB) which mostly affects people in developing countries. According to the WHO’s global TB report of 2018, TB is one of the top 10 causes of death and in 2017 there were about 1.6 million TB deaths and 10 million infections [1]. Emerging drug resistance hinders the progress and efforts to control this disease [2]. Among all drugs available, pyrazinamide (PZA) is an important anti-tuberculosis drug against the dormant or semi-dormant latent stage of MTB [3]. Despite its importance, PZA is also responsible for a relevant proportion of treatment abandons because of side effects [4]. In Peru, over 30% of multidrug-resistant (MDR) TB strains are also resistant to PZA [5]. Moreover, in other countries exists a large prevalence of PZA resistance [6].

The mechanism of action and resistance to PZA in MTB is not entirely understood [3]. PZA, the pro-drug, enters the cytoplasm of MTB by passive diffusion and is hydrolyzed into pyrazinoic acid (POA) by the enzyme pyrazinamidase (PZAse), encoded by the *pncA* gene [7]. POA, the active drug, accumulates in the cytoplasm and enters a cycle of entry and exit from MTB aided by an efflux pump not yet identified. Outside, in an acidic environment, POA is protonated and when re-enters to the cytoplasm, releases the protons and causes membrane disruption and cellular damage [8].

The major mechanism of PZA resistance, according to several studies, is the loss of PZAse activity due to mutations in the *pncA* gene, suppressing its ability to hydrolyze PZA [9–12]. Mutations directly affecting the active site (AS) (Cys138, Asp8, and Lys96), or the metal coordination site (MCS) (Asp49, His51, His57, and His71) are more likely to impair PZAse function [13]. Current knowledge of *pncA* gene sequences has shown that PZA resistant strains are associated with *pncA* mutations scattered throughout the entire gene which deplete the PZAse function [10,14–16]. It is important to highlight that a failure in the PZAse function strictly causes resistance to PZA.

The current gold standard test to detect PZA resistance is the colorimetric MGIT 960-TB liquid culture. Alternate assays comprise the MODS [17] and MODS-PZA [18] methods, based on microscopic observation and the colorimetric Wayne test and variants like the reported by Aono *et al*., 2018 [19] or Alcántara *et al*., 2019 [20]. The latter detect expelled POA in the extracellular environment, a biomarker of PZA resistance [14,21], and indirectly measures the PZAse activity [20]. Nevertheless, the high cost, duration, or lack of reproducibility of these tests limit their use. Therefore, an accurate, simple, and fast test to determine PZA resistance is required to prevent the unnecessary use of PZA during anti-tuberculous treatment.

Given the current extensive use of next-generation sequencing (NGS), it has been proposed as an alternative to detect PZA-resistance by the detection of mutations in the *pncA* gene [22–24]. Nowadays, the sequencing of this gene is more affordable, numerous studies have reported *pncA* polymorphisms associated with PZA resistance with high accuracy [10,22,25–27]. Nevertheless, most of them are focused on susceptible/resistant binary prediction instead of predicting PZAse function and PZA resistance level. The main limitation of this approach is the fact that not every *pncA* mutation impairs the PZAse enzymatic function. To overcome this limitation, a method to predict the enzymatic function based only on the *pncA* sequence is needed.

Several studies have addressed the prediction of the enzymatic function of enzymes based on the predicted structure of mutated enzymes [28–34]. The approach of these studies used several features as predictors, including electrostatic potentials used in quantitative structure-activity relationships (QSAR) [35–37] and geometrical descriptors that are measurements of distances between specific points of interest and indirectly involve short and long-range interactions between residues [38–40].

Amino acidic mutations affecting protein function may alter the protein structural stability [31,41,42]. In particular, structural stability has been correlated with the enzymatic function in PZAse [43]. This property could be studied by several techniques like molecular dynamics (MD) simulations, using the root-mean-square fluctuation (RMSF) of residues, to find unstable regions with a high degree of movement and flexibility [44,45]. In PZAse the most unstable region is the flap region, a loop from His51 to His71 [45–49], which is the lid of the PZA/ Fe^2^ binding cavity [13], and alterations of its stability have been reported in mutated PZAses associated with PZA resistance [46–49].

An alternative, faster and reliable method to analyze protein stability, is the Normal Mode Analysis (NMA), which is based on the protein structure [50,51]. NMA considers oscillating movements that describe relevant motions of small amplitude at the atomic or aminoacidic level [52,53]. It assumes that proteins oscillate harmonically around a given conformation [54] and calculates the normal modes of vibrations to describe the overall motion. From this, fluctuation scores for each position in the protein are calculated [55].

In this study, we developed statistical models to predict PZAse function, based on structural stability, physicochemical, and geometrical descriptors inferred from 3D modeled structures of PZAse based only on the mutated sequence. Multiple log-linear regressions and 6-fold cross-validation were used to get the best linear predictors and to verify robustness.

## Methods

### Cloning and expression of PZAse

The sequences of 35 pncA mutant genes from clinical strains, among the H37Rv wild-type were cloned and expressed in *Escherichia coli* LEMO21 as reported before [7]. Briefly, each *pncA* gene sequence was amplified by PCR from genomic DNA using primers containing restriction sites for *Nco*I and *Xho*I and inserted into pET28a plasmid containing a COOH-terminal 6-His tag. Both PCR product and pET28a plasmid were digested with *Nco*I and *Xho*I, ligated and transformed into *E*. *coli* LEMO21. Proteins were purified by affinity chromatography using a HisTrap HP column (Novagen).

### Kinetic parameters of recombinant PZAses

PZAse kinetic parameters were calculated using the hydrolysis reaction of PZA. Briefly, PZA was used from 0 to 5 mM and incubated with 1 μM PZAse in 50 mM sodium phosphate pH 6.5. To prevent the hydrolysis of more than 10% of the initial PZA, an incubation period of 1 min was used. It was increased to 2 h for mutants with very low activity. 10 μL of 20% Ferrous ammonium sulfate was added, followed immediately by adding 445 μL of 0.2 mM glycine-HCl (pH 3.4) to stop the reaction. Precipitates were removed by centrifugation (11,000 rpm for 5 min). Absorbance (OD) was measured at 450 nm in a 96-well plate using 200 μL of reaction. The amount of POA produced was estimated by interpolation in a standard curve of known concentrations. Each recombinant PZAse was tested at least 5 times, and at least 2 different groups of recombinant proteins were analyzed for each strain. Finally, we estimated k_cat_, K_M_, efficiency, and activity as before [7].

To make the measurements of the kinetic parameters comparable between the two batches of recombinant proteins, we normalized each kinetic parameter by dividing its value by the one of the wild type (WT) H37RV PZAse of the corresponding batch.

### Theoretical structural modeling of mutated PZAses

All mutants were modeled using the protein modeling server SWISS-MODEL [56] with the crystal structure of the WT PZAse available in Protein Data Bank (PDB ID: 3PL1, 2.2 Å resolution) as a template.

### Stability analysis of amino-acidic fluctuations by NMA

We evaluated structural stability using the fluctuation scores for each amino acid position calculated by NMA [57]. Briefly, a potential energy function at residue level is constructed for each structure using an elastic network model that connects all the Cα’s of residues that interact with each other with a spring. The original code was modified to consider connected the Cα’s of pairs of residues for which the distance between any main chain or side chain atoms was less than 5 Å. All Cα- Cα pairwise forces were modeled as a harmonic oscillator with the same spring constant. Then, the modes are calculated by solving the following equation:

HX=DTX (1)

Where H is the Hessian matrix of the potential energy function, T the kinetic energy, X the eigenvectors, and D a diagonal matrix containing the eigenvalues [57]. The frequency of oscillation of each normal mode is related to the eigenvalues and is the same for all the residues in the respective normal mode. Each normal mode has an associated eigenvector of length equal to the number of residues (or 3N). The components of the eigenvector corresponding to the amplitude and the direction of movement of each Cα.

We considered 40 modes, from the 7^th^ up to the 46^th^ eigenvalues. The first six were left out because they correspond to the 3 translations and 3 rotations and were almost zero. After that, the fluctuations for each amino acid in each mutated structure were calculated by the equation [55]:

$${<x}_{i}^{2}>=\frac{k_{B}T}{m_{i}}\sum_{j=i}^{n}\frac{a_{ij}^{2}}{w_{j}^{2}}$$ (2)

Where k_B_ is the Boltzmann constant, T the absolute temperature, m_i_ the mass of residue i, n the number of modes, a_ij_ the eigenvector for residue i in mode j, and w_j_ the square root of the eigenvalue for mode j. A final dataset of 185 variables for each structure was produced.

### Stability analysis of amino-acidic RMSF by MD

As an alternative approach to stability calculated by NMA, molecular dynamics (MD) simulations were performed on each PZAse structure. For this purpose, the software GROMACS version 2018.3 [58] in GPU configuration was used to simulate the behavior in a solvent of the mutated structures modeled with SWISS-MODEL [56] and the WT-PZAse (PDB ID: 3PL1) [13].

During the simulation, topologies were generated with the PDB2GMX module, using the OPLS-AA/L all-atom force field [59]. Additionally, each structure was centered in a 10 Å cubic box, with periodic boundary conditions. Water molecules from the SPC/E model [60] were used for solvation, and charges were neutralized by adding Na^+^ and Cl^-^ ions.

Afterward, energy minimization of the system was performed using the steepest descent algorithm with 50 000 steps until obtaining energy lower than 1000 Kcal mol^-1^. The temperature and the pressure of the system were equilibrated by using NVT and NPT assembling, respectively. In the first stage, the temperature was equilibrated to 310.15 K and constantly maintained with the Berendsen thermostat [61] for 2 ns. In the second stage, the system’s pressure was equilibrated to 1 bar and constantly maintained with the Parrinello-Rahman barostat [62,63] for 4 ns. Finally, the simulation time was 500 ns, with 2 fs integration step and constant pressure and temperature conditions, using the integration algorithm leap-frog [64]. For generating the trajectories, the LINCS algorithm [65] was used to restrict interactions during equilibrium, while the Particle-Mesh Ewald algorithm [66] was used to restrict the long-range ionic interactions.

The trajectories obtained from each simulation were centered and used to calculate the root-mean-square deviation (RMSD) of the protein of each structure. Likewise, the trajectories corresponding to the last 100 ns of each molecular dynamics (400 to 500 ns) were extracted to calculate the root-mean-square-fluctuations (RMSF) of the backbone. The data was extracted in *.XGV format and RMSD and RMSF plots were generated using an *in-house* python script using the Matplotlib library [67]. A final dataset of 185 RMSF variables for each structure was generated.

### Calculation of physicochemical descriptors

The physicochemical descriptors evaluated were the differences in electrostatic potential (EP) between residues from each mutated protein and residues from the WT-PZAse and the sum of all differences in electrostatic potential in the entire protein. The MutantElec server [68] was used to calculate EPs, this server uses an Adaptive Poisson-Boltzmann Solver (APBS) to analyze the effect of solvents in proteins and as input requires the dielectric constant of protein (Ɛ_protein_) and water (Ɛ_water_) at a fixed temperature. We assumed a value of Ɛ_protein_ of 4 as a mean for proteins and calculated Ɛ_water_ = 74.1522 at *T* = 37°C (which represents lung temperature) as previously reported [69]. A final dataset of 185 physicochemical variables for each structure was generated.

### Calculation of geometrical descriptors for WT and mutated PZAses

For every mutated PZAse structure, we calculated 4 references as described previously [38] (S2 Fig): (i) Point B: the barycenter of the AS (Asp8, Lys96, and Cys138) and the MCS (Asp49, His51, and His71 excluding His57). (ii) Point P: the point of projection of the Cα of the residues on the plane formed by the trio of amino acids (AS/MCS). (iii) Point I: the point of intersection between the resulting vector (Vr) of each amino acid regarding the C_α_ and the plane formed by the trio of amino acids (AS/MCS). (iv) Point T: the barycenter of the MCS including His57.

Using the points of interest, 24 different descriptors were calculated on each of the 185 aminoacidic positions of PZAse resulting in 4440 variables. The exact meaning of each geometrical descriptor is available in the S1 Appendix.

### Construction and validation of predictive linear models

Since kinetic parameters of enzymes are strictly positive variables and tend to follow a log-normal distribution in nature [70], we assumed them as log-normally distributed. The following transformation was used to go from the mean of the kinetic parameters to the mean of the logarithmic kinetic parameters:

$$\bar{{log}_{10}(x)}=\log_{10}\left(\frac{{\bar{x}}^{2}}{\sqrt{{\bar{x}}^{2}+\sigma_{x}^{2}}}\right)$$ (3)

Where $\bar{x}$ refers to the mean value of the respective kinetic parameter, and $\sigma_{x}^{2}$ to its variance. We worked with these estimates to construct the log-linear models. Each kinetic parameter was combined with the stability, physicochemical and geometrical datasets separately. Observations with a missing value in the respective kinetic parameter were removed from each dataset. Then, covariates with missing values or variance equal to zero were dropped.

To avoid correlation between covariates, simple log-linear regressions between the kinetic parameter and the rest of covariates were performed for each dataset. The p-value for each regression was used to sort covariates in ascending order. Then, we went through the sorted list and calculated Pearson’s correlation coefficient between the top covariate and the rest of the covariates under it. If the correlation coefficient was greater than 0.8, the covariates down in the list were removed from the dataset and the procedure continue with the next covariate in the reduced list until the last covariate is reached.

Once the datasets were reduced, covariates were selected using a stepwise Akaike’s information criterion (AIC) forward regression until having 10 covariates. The final individual models include the best combination of 6 out of the 10 selected covariates that display the highest adjusted R^2^. The weighted models were constructed for each kinetic parameter using the fitted values of their respective individual models as covariates.

To prevent overfitting, we performed a repeated 6-fold cross-validation for the weighted models and the individual models of stability, physicochemical, and geometrical descriptors using the R’s package Caret [71]. The root-mean-square error (RMSE) calculation was used to evaluate the predictive ability of each model. To have a clear idea of the distribution of RMSE values of our models, we performed a repeated 6-fold cross-validation 1000 times and compare the distribution of the RMSE for individual models with the one produced by models with random descriptors (random models). For the weighted model, the RMSE distribution was compared against the one produced by the individual models.

## Results

### Kinetic parameters of recombinant PZAses

The kinetic parameters measured for the 35 mutated and WT PZAses revealed a wide range of variation (Table 1 and S1 Table). Due to experimental error, the WT H37Rv PZAse showed slightly different estimates of the kinetic parameters between the two batches of proteins produced. To prevent a batch-bias, we expressed all the kinetic parameters as the corresponding percentage of the WT-PZAse within each batch and worked with these new variables defined as Relative-k_cat_, Relative-K_M_, Relative-efficiency, and Relative-activity. The relative kinetic parameters revealed a high Pearson correlation coefficient (R) between k_cat_ and efficiency (R = 0.787), k_cat_ and activity (R = 0.947) and efficiency and activity (R = 0.865), while K_M_, was neither well correlated with k_cat_ (R = 0.072), efficiency (R = -0.286), nor activity (R = -0.160).

**Table 1:** Kinetic parameters of two batches of mutated PZAses from *M*. *tuberculosis*.

	Mutant	k_cat_ (min^-1^)	Relative k_cat_ (%)	K_M_ (mM)	Relative K_M_ (%)	Efficiency (mM^-1^min^-1^)	Relative Efficiency (%)	Activity (μmol POA min^-1^ mg^-1^ PZAse)	Relative Activity (%)
**Batch 1**	**WT**	1005.410	100.000	1.240	100.000	806.600	100.000	38.400	100.000
	A102V	1084.220	107.839	1.581	127.479	685.890	85.035	34.316	89.364
	C14G	25.110	2.497	1.327	107.014	18.920	2.346	0.902	2.350
	**D12A**	245.170	24.385	0.990	79.839	248.790	30.844	9.240	24.063
	**D12G**	368.460	36.648	0.550	44.355	716.830	88.871	14.000	36.458
	D136G	490.270	48.763	3.680	296.774	133.030	16.493	12.340	32.135
	**D49N**	1.530	0.152	3.090	249.194	0.550	0.068	0.045	0.117
	F58L	355.880	35.397	1.297	104.627	274.300	34.007	14.132	36.801
	**F94L**	712.920	70.908	2.000	161.290	348.600	43.218	21.190	55.182
	**G24D**	100.130	9.959	0.420	33.871	236.200	29.283	4.280	11.146
	**G78C**	105.160	10.459	1.070	86.290	97.650	12.106	6.960	18.125
	**H51R**	0.170	0.017	1.410	113.710	0.120	0.015	0.006	0.016
	H57R	12.020	1.196	1.099	88.609	10.940	1.356	0.505	1.316
	H71Y	69.840	6.946	10.657	859.464	9.320	1.155	1.425	3.710
	**K48T**	241.820	24.052	0.440	35.484	551.200	68.336	10.450	27.214
	**L116P**	1324.500	131.737	1.560	125.806	847.660	105.091	50.150	130.599
	L4S	0.001	0.000	0.201	16.173	0.003	0.000	0.015	0.039
	P54L	141.195	14.044	1.001	80.763	140.989	17.479	5.529	14.398
	P62L	203.859	20.276	0.779	62.802	261.782	32.455	10.129	26.378
	Q10P	37.665	3.746	0.650	52.431	57.932	7.182	1.978	5.151
	R29P	59.000	5.868	0.225	18.117	262.881	32.591	2.761	7.189
	**T135P**	0.450	0.045	0.930	75.000	-	-	0.020	0.052
	T142A	0.040	0.004	1.130	91.090	0.040	0.005	0.002	0.005
	T160K	3.500	0.348	13.630	1099.194	0.260	0.032	0.038	0.100
	**T76P**	202.980	20.189	0.310	25.000	650.330	80.626	8.990	23.411
	V139A	628.710	62.533	25.422	2050.140	24.730	3.066	3.455	8.997
	W119L	17.090	1.700	1.234	99.508	13.850	1.717	0.883	2.299
	**Y34D**	386.400	38.432	0.830	66.935	460.190	57.053	20.580	53.594
	Y64D	1357.250	134.995	1.548	124.870	876.550	108.672	57.830	150.598
**Batch 2**	WT	739.480	100.000	1.162	100.000	648.776	100.000	27.607	100.000
	A171T	408.035	55.179	1.501	129.177	283.986	43.773	16.422	59.484
	A46V	244.662	33.086	1.121	96.436	260.875	40.210	11.433	41.411
	L172P	11.730	1.586	1.469	126.390	10.877	1.677	0.500	1.811
	M175V	199.743	27.011	2.260	194.516	96.922	14.939	5.776	20.921
	P62R	206.273	27.894	1.469	126.430	141.472	21.806	7.889	28.574
	V125F	609.467	82.418	1.678	144.418	407.886	62.870	19.996	72.428
	V180F	474.632	64.185	6.309	542.873	149.531	23.048	9.104	32.975

The relative kinetic parameters are expressed as percentages of the corresponding WT-PZAse kinetic parameters. The mutants previously reported by our group (Sheen et al., 2009) are in bold. For the mutant T135P, the experimental measurements of efficiency are not available.

### Structural modeling of mutated PZAses and stability, physicochemical, and geometrical structural analysis

The modeled structures of the mutated PZAses, showed an average root-mean-square deviation (RMSD) of 0.094 Å, at all-atoms level, compared to the WT-PZAse crystal structure (PDB: 3PL1) (S1 Fig).

We estimated, first, 4440 geometrical descriptors of distances between specific points of interest related to the AS/MCS and all the residue positions of the protein (S1 Dataset). A more detailed explanation of the geometrical meaning of these descriptors is available as supplementary material (S2 Fig and S1 Appendix). Second, 185 physicochemical descriptors that include the difference in electrostatic potential per residue (DEPR) against the WT-PZAse and the global difference in electrostatic potential (GDEP) (S2 Dataset and S3 Fig). Third, 185 stability descriptors of amino-acidic fluctuations calculated by NMA that describe the degree of fluctuation of each residue averaged over 40 normal modes of vibration (S3 Dataset and S4 Fig). Alternatively, we performed MD simulations of 500 ns (S5 Fig) on each structure to calculate the RMSF of the protein backbone and to use it as an alternative descriptor of stability for each position; other 185 descriptors were generated (S4 Dataset and S6 Fig).

**Fig 1:**
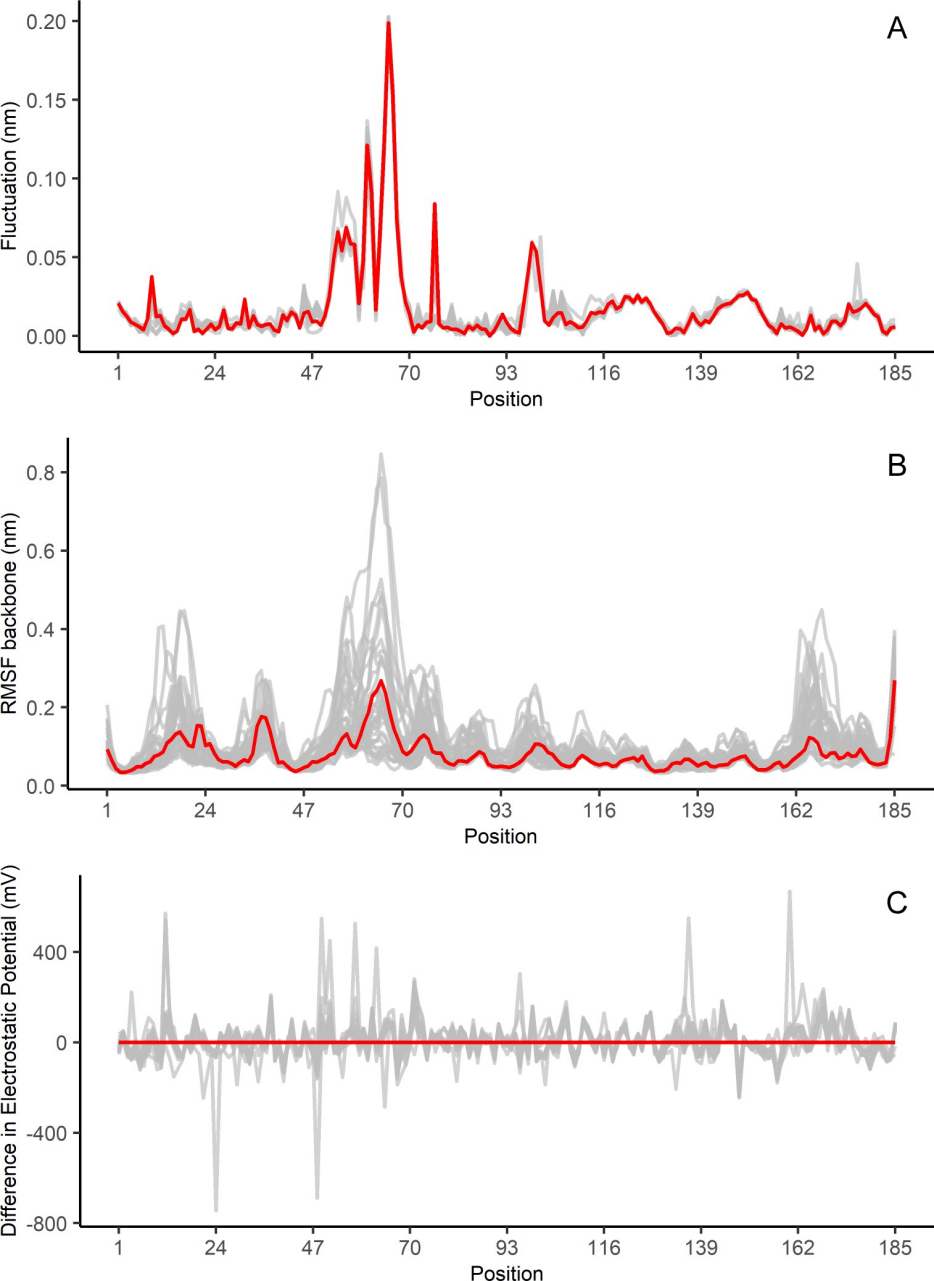
Fluctuation, RMSF, and DPER profiles for PZAses obtained from normal mode analysis (NMA), molecular dynamics (MD), and MutantElec server. (A) Fluctuation profiles. The red trace represents the WT profile. Low variability is observed for the fluctuation score along with all the positions. The overall shape of the profile is maintained along with the flap region spanning from His51 up to His71 being the most flexible part of PZAse. (B) RMSF profiles. The red trace represents the WT profile. A higher variability than in the case of NMA fluctuations is observed. The flap region going from His51 up to His71 shows the maximum values for RMSF. Additionally, two other regions with high RMSF are observed near the N and C-terminal of the protein. (C) DEPR profiles. The red trace represents the WT profile. Differences greater than ±400 mV are observed as extreme peaks, some of them belonging to the flap region of PZAse.

For the profile of stability per position, although sharing the same overall shape with the highest peak in the flap region going from His49 until His71, the fluctuations calculated with NMA (Fig 1A) showed a lower variability than the RMSF calculated with MD (Fig 1B). Concerning the DEPR, the profile also exhibits a high variability compared to the WT-PZAse (Fig 1C), illustrating the effect in electrostatic potential introduced by missense mutations.

Comparing the different PZAses with a relative-k_cat_ higher or equal than 50%, against the mutated PZAses with a relative-k_cat_ lower than it, we found almost similar patterns of stability descriptors in both groups for NMA (S7A Fig and S7B Fig) and MD (S7C Fig and S7D Fig) approaches. In the case of physicochemical descriptors, a higher dispersion with outliers is observed for the group with relative-k_cat_ less than 50 (S7E Fig and S7F Fig). After performing a two-sided Mann-Whitney-U non-parametric test for stability descriptors derived from NMA, we found statistically significant differences in the positions Leu27 (P-value = 0.022), Pro70 (P-value = 0.011), Tyr103 (P-value = 0.014), Val109 (P-value = 0.029), and Ser179 (P-value = 0.024). On the other hand, no statistically significant differences were found in any position for stability descriptors derived from MD simulations. Concerning DEPR, the positions Val45 (P-value = 0.026), Ala46 (P-value = 0.047), Asp49 (P-value = 0.032), His51 (P-value = 0.029), His57 (P-value = 0.029), Ser65 (P-value = 0.047), Arg121 (P-value = 0.024), Val131 (P-value = 0.047), and Asp166 (P-value = 0.016) showed a significant difference. Interestingly, three out of four residues of the MCS are included in that list. However, after correcting for multiple testing using the Bonferroni correction or the Benjamini-Hochberg procedure for an FDR of 10%, any significant position is found in any dataset.

### Log-linear models for kinetic parameters: K_cat_, K_M_, efficiency, and activity

For each kinetic parameter, six log-linear models were constructed. Four of them correspond to individual linear models of six covariates of only physicochemical, geometrical, stability (NMA) or stability (MD) descriptors. The other two are weighted log-linear models built over the fitted values of the individual physicochemical, geometrical, and stability (NMA) or stability (MD) models.

**Fig 2:**
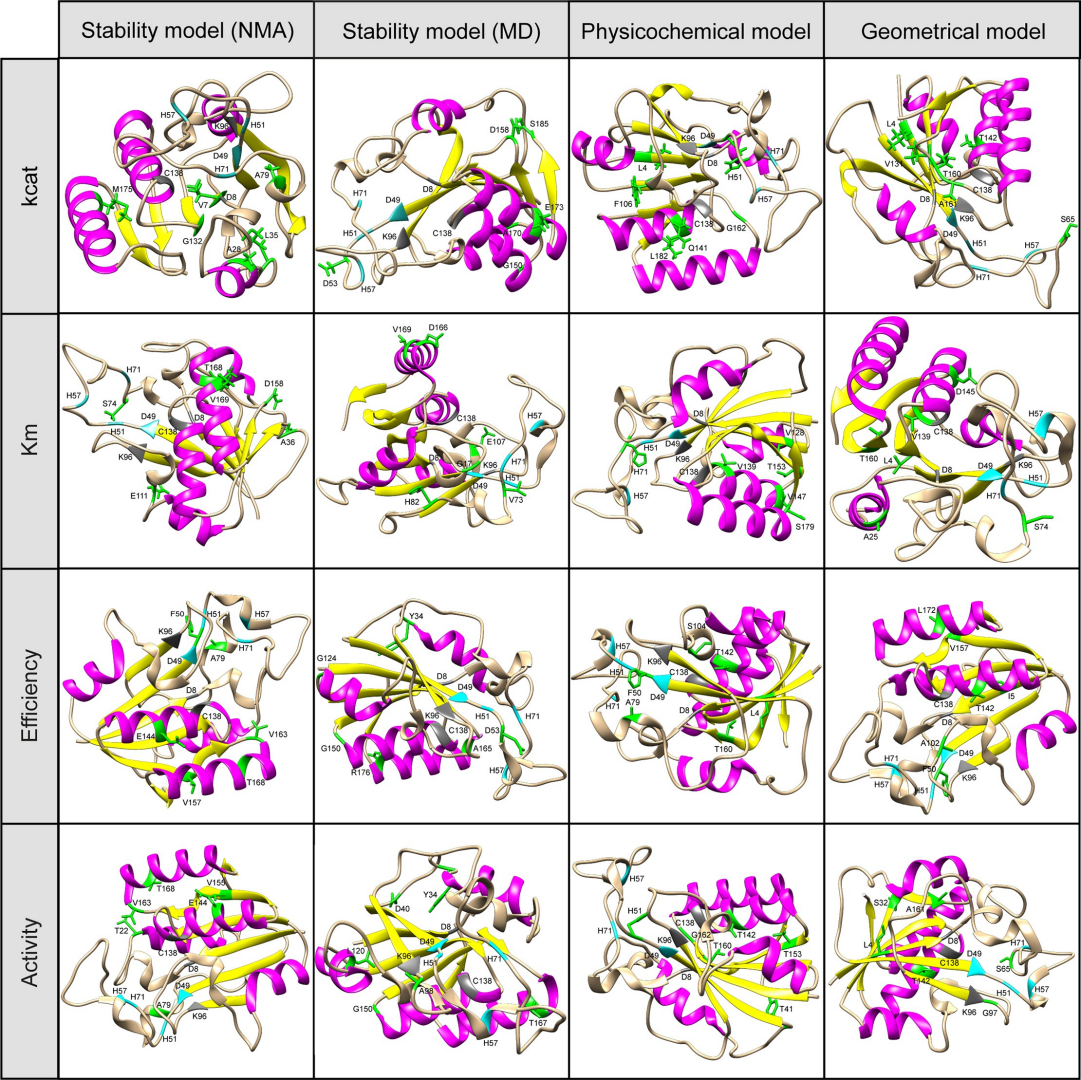
Structures of WT-PZAse highlighting the positions associated with the selected descriptors for individual models. In green, residues from each descriptor of the model; in gray, residues from the AS; in sky blue, residues from the MCS; in pink, residues from alpha-helix and in yellow, residues from beta-strands.

The selected descriptors in individual models involve different positions along PZAse (Fig 2 and Table 2). The Pearson correlation between descriptors in individual models is less than 0.8. We compared the distribution of the root-mean-square error (RMSE) of prediction calculated by 6-fold cross-validation for individual models against one of the random models of the same number and kind of descriptors. We found that individual models always have a mean RMSE lower than the random models, although the amount of the difference varies between different models (S8 Fig–S23 Fig).

**Table 2:** Selected descriptors for individual models of each kinetic parameter.

		Kinetic parameters			
		k_cat_	K_M_	Efficiency	Activity
**Selected descriptors**	**Stability model (NMA)**	Fluctuation Leu 35	Fluctuation Asp 158	**Fluctuation Thr 168**	**Fluctuation Thr 168**
		Fluctuation Met 175	Fluctuation Ala 36	**Fluctuation Ala 79**	**Fluctuation Ala 79**
		Fluctuation Gly 132	Fluctuation Glu 111	Fluctuation Val 157	**Fluctuation Glu 144**
		Fluctuation Val 7	Fluctuation Val 169	Fluctuation Phe 50	**Fluctuation Val 163**
		**Fluctuation Ala 79**	Fluctuation Ser 74	**Fluctuation Val 163**	Fluctuation Thr 22
		Fluctuation Ala 28	**Fluctuation Thr 168**	**Fluctuation Glu 144**	Fluctuation Val 155
	**Stability model (MD-RMSF)**	**RMSF Gly 150**	RMSF Gly 17	**RMSF Gly 150**	RMSF Asp 40
		**RMSF Gly 124**	RMSF Glu 107	**RMSF Gly 124**	**RMSF Gly 150**
		**RMSF Asp 53**	RMSF Val 169	RMSF Arg 176	RMSF Leu 120
		RMSF Glu 173	RMSF Asp 166	**RMSF Asp 53**	**RMSF Tyr 34**
		RMSF Ser 185	RMSF Val 73	**RMSF Tyr 34**	RMSF Thr 167
		RMSF Asp 158	RMSF His 82	RMSF Ala 165	RMSF Ala 98
	**Physicochemical model**	**DEPR Leu 4**	DEPR Val 139	**DEPR Leu 4**	**DEPR His 51**
		**DEPR His 51**	**DEPR Thr 153**	DEPR Phe 50	**DEPR Thr 160**
		DEPR Gln 141	DEPR Val 128	**DEPR Thr 160**	**DEPR Gly 162**
		DEPR Phe 106	DEPR Ser 179	DEPR Ala 79	**DEPR Thr 153**
		DEPR Leu 182	DEPR His 71	**DEPR Thr 142**	**DEPR Thr 142**
		**DEPR Gly 162**	DEPR Val 147	DEPR Ser 104	DEPR Tyr 41
	**Geometrical model**	**B_I (AS) Leu 4**	C_C138 Ser 74	**B_I (AS) Leu 4**	**B_I (AS) Thr 142**
		**B_I (AS) Thr 142**	**B_I (AS) Leu 4**	**B_I (AS) Thr 142**	P_C (MCS) Gly 97
		**C_C138 Ala 161**	B_P (MCS) Thr 160	**C_C138 Val 157**	**B_I (AS) Leu 4**
		**I_P (MCS) Ser 65**	C_K96 Ala 25	C_C138 Ala 102	**C_C138 Ala 161**
		C_D49 Val 131	C_D49 Asp 145	P_T (MCS) Leu 172	**I_P (MCS) Ser 65**
		I_C (MCS) Thr 160	B_I (AS) Val 139	I_P (MCS) Phe 50	**C_C138 Val 157**

The descriptors selected in more than one model are in bold. The meaning of geometrical descriptors is available at S1 Appendix.

An interesting result when comparing the alternative stability models is that RMSF descriptors generated by MD simulations always produce models with higher R^2^ and lower mean RMSE, probably due to the higher degree of variability between structures detected with this approach.

The weighted models have three covariates, representing the predictions of the stability, physicochemical and geometrical models for k_cat_ (S24 Fig and S25 Fig), K_M_ (S26 Fig and S27 Fig), efficiency (S28 Fig and S29 Fig) and activity (S30 Fig and S31 Fig). The coefficients for each covariate assign a weight to the prediction of the corresponding individual model. Overall, the six models for K_M_ have the lowest mean RMSE, always below 0.4. For k_cat_, efficiency, and activity only the weighted models have a mean RMSE lower than 0.51. Regarding their individual models, the mean RMSE ranges from 0.906 to 1.37. A summary of the statistics of all the models for each kinetic parameter including R^2^, adjusted R^2^, p-value and mean RMSE is displayed in Table 3.

**Table 3:** Summary statistics of models.

	**k**_**cat**_				**K**_**M**_			
	p-value	R^2^	Adjusted R^2^	<RMSE>	p-value	R^2^	Adjusted R^2^	<RMSE>
**Weighted Model (NMA)**	7.44E-16	0.897	0.887	0.5057	6.13E-14	0.864	0.851	0.1586
**Weighted Model (MD)**	8.70E-18	0.922	0.914	0.4299	3.20E-14	0.869	0.857	0.1562
**Stability model (NMA)**	7.76E-03	0.432	0.314	1.2514	6.93E-04	0.531	0.434	0.3441
**Stability model (MD)**	4.52E-05	0.619	0.54	1.0708	8.67E-06	0.662	0.593	0.2764
**Physicochemical model**	1.85E-08	0.784	0.739	0.9215	3.54E-08	0.773	0.727	0.247
**Geometrical model**	2.26E-11	0.866	0.838	1.3692	4.11E-08	0.771	0.724	0.265
	**Efficiency**				**Activity**			
	p-value	R^2^	Adjusted R^2^	<RMSE>	p-value	R^2^	Adjusted R^2^	<RMSE>
**Weighted Model (NMA)**	4.34E-17	0.92	0.912	0.4265	3.77E-16	0.901	0.892	0.4013
**Weighted Model (MD)**	6.80E-19	0.939	0.933	0.3707	4.45E-16	0.9	0.891	0.4001
**Stability model (NMA)**	4.51E-03	0.467	0.353	1.2189	2.05E-02	0.384	0.257	1.0487
**Stability model (MD)**	9.96E-06	0.671	0.601	0.9883	7.61E-05	0.603	0.521	0.9065
**Physicochemical model**	4.01E-10	0.845	0.812	1.1132	4.02E-08	0.771	0.724	0.91
**Geometrical model**	1.16E-12	0.899	0.877	1.2126	1.68E-08	0.785	0.741	1.0839

P-value refers to significance; R^2^ is the coefficient of determination and <RMSE> is the mean of Root-Mean-Square Error.

### Biological significance of the selected descriptors

**Fig 3:**
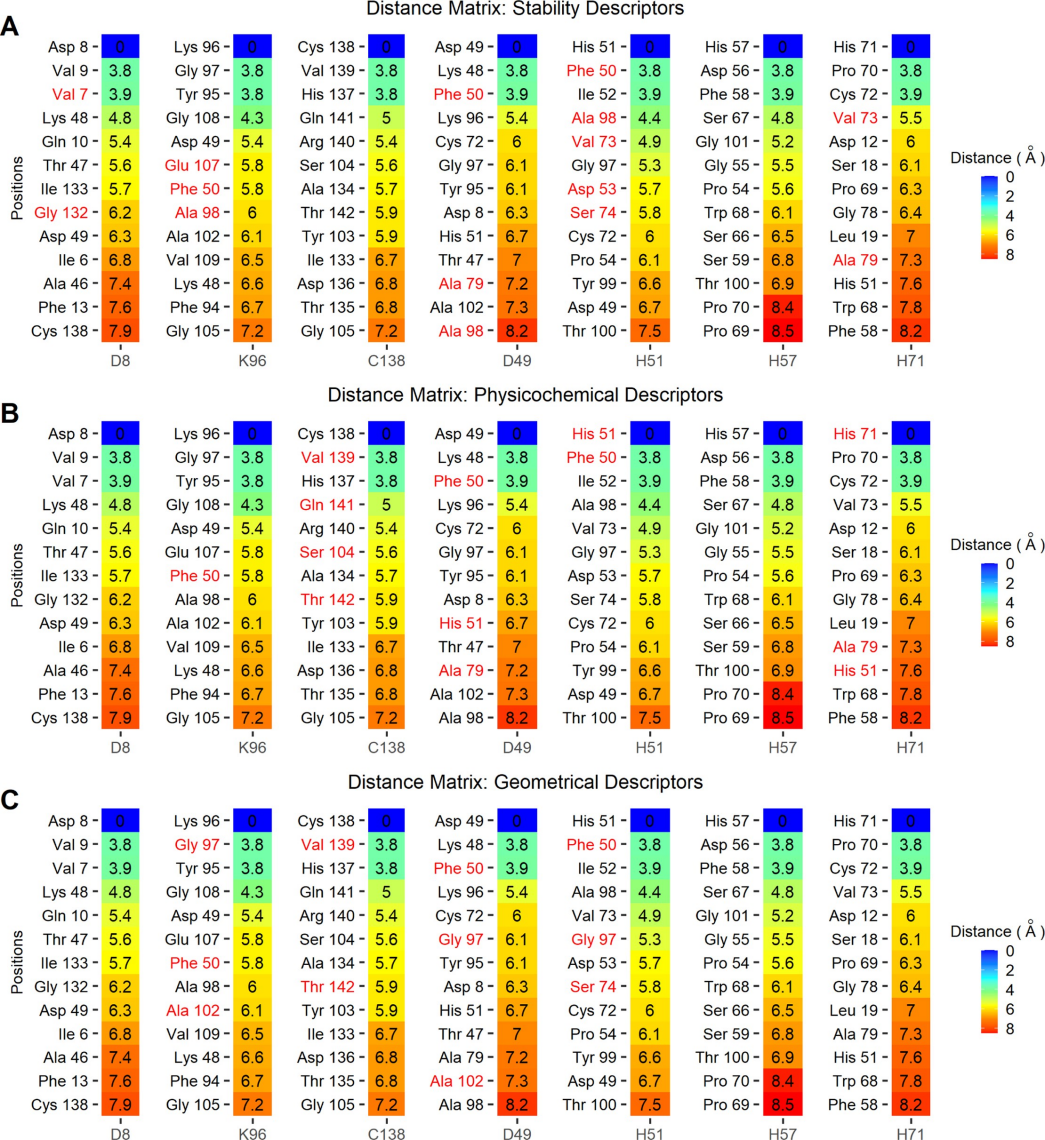
Distance matrix of WT-PZAse residues position against AS and MCS. The distance between pairs of C_α_ is sorted ascendingly. The matrix shows the 13 positions closest to the residues of the AS and MCS. The residues whose position is included in the selected descriptors are depicted in red for (A) stability models (NMA and MD), (B) physicochemical models, and (C) geometrical models.

Although the descriptors selected by individual models are not necessarily involved in the mechanism of action, we found that some positions associated with the selected descriptors are relatively close to the AS or the MCS. Thus, the distances between the C_α_ of each residue in the WT-PZAse and the C_α_ of the residues of the AS and MCS were calculated and sorted to highlight the closest positions to these points (Fig 3 and S2 Table). To select the closest positions a cutoff of 7 Å was arbitrarily chosen. The residues with positions included in the selected descriptors close to at least one of the residues of the AS or MCS are Phe50, Ser74, Gly97, Ala102, Val139, and Thr142 for the geometrical models, Phe50, His51, His71, Ser104, Val139, Gln141, and Thr142 for the physicochemical models, and Val7, Phe50, Asp53, Val73, Ser74, Ala98, Glu107, and Gly132 and for the stability models (NMA and MD). These positions reflect the importance of the stability, electrostatic potential, and geometrical distances near the AS/MCS, while the rest of positions the effect of long-range interactions in the kinetics parameters of PZAse.

## Discussion

In this study, we developed a method to predict PZAse kinetic parameters (*k*_*cat*_, *K*_*m*_, efficiency, and activity) based on recognizing a mutation in the *pncA* coding gene sequence. Stability, physicochemical, and geometrical descriptors got from the modeled 3D structure were used to build multiple log-linear models to predict the kinetic parameters. Our weighted models can predict the experimentally measured enzymatic kinetic parameters with high accuracy, with adjusted R^2^ values ranging from 85.1% to 93.3%. These models confirmed to be robust after a 6-fold cross-validation analysis, with the models for activity and *K*_*M*_ having the lowest mean RMSE.

The kinetic parameters studied belong to two groups. A group related to the catalytic activity (k_cat_, efficiency, and activity) and a group related to substrate affinity (K_M_). Even though efficiency depends inversely on K_M_, it showed to be highly correlated with k_cat_ and activity. While a low value for the group related to catalytic activity has a deleterious effect in the enzymatic reaction, the opposite is true for the group related to substrate affinity, where a high value of K_M_ implies that a higher concentration of substrate is needed to have the enzyme working at its maximum velocity. In our data, the distribution of K_M_ showed that most mutants have similar values to the WT-PZAse, while only six mutants (D49N, D136G, V180F, H71Y, T160K, and V139A) have values of K_M_ 2-fold greater than the WT. For k_cat_, efficiency, and activity, several mutants have very low values, showing that missense mutations affect catalytic activity rather than substrate affinity most of the time.

Regarding the physicochemical descriptors, the cavity formed by the AS and the MCS of PZAse is rich in residues with a negative charge at neutral pH. At physiological pH, POA and PZA are negatively charged (pKa 2.9 and 0.5 respectively [72]. Based on this evidence, we hypothesize that an increase in the DEPR (compared to the WT-PZAse) at positions close to this cavity will increase the affinity for PZA of the enzyme but decrease the release rate of the POA. This is important to consider, since PZA is a prodrug and does not act as an inhibitor, but needs to be hydrolyzed into POA and then released from the PZAse to perform its lethal effect.

Theoretically, increasing or decreasing DEPR may eventually cause enzymatic dysfunction. Interestingly, variation in the DEPR of positions His51 and His71 from MCS are negatively correlated with k_cat_, and K_M_, respectively, showing that a higher DEPR will reduce the catalytic activity (slower release) and the dissociation constant (stronger interaction, lower amounts of substrate needed to saturate the enzyme). Also, for DEPR, three positions belonging to the MCS (Asp49, His51, and His57) showed a statistically significant difference, suggesting that the difference in electrostatic potentials could be informative for classifying functional and non-functional PZAses. To confirm this hypothesis, further experimental studies are required to evaluate a larger number of mutants.

Our stability data from NMA and MD approaches shows that the fluctuation profile is overall conserved among PZAses, with the flap region from His51 to His71 being the most flexible part of the enzyme, as previously reported [45–49,73]. This loop is in the binding’s lid cavity and contributes to the ability of PZA to enter the PZAse active site cavity [13]. At the prediction level, stability predicted by MD simulations performed better than that predicted by NMA. This could be because of the different degrees of variation detected by NMA and MD. While NMA detects almost no variation in the fluctuation profiles of PZAses (S4 Fig), MD allows a better resolution of the effect of punctual mutations in the stability of PZAse. However, in terms of prediction time, NMA based stability and weighted models will provide much faster estimates than MD based models [51].

After dichotomizing the PZAses in two groups using a value of relative-k_cat_ of 50% as a cutoff (high relative-k_cat_ group and low relative-k_cat_ group), we did not find statistically significant differences in the RMSF values, but for the fluctuation scores, only one position from the flap region (Pro70) was significant. Although previous studies showed that PZAse function decreases with an increment in the RMSF of the flap region, [42–46,74], we did not find statistical evidence for that. This apparent discrepancy may occur because other studies only compared the fluctuation profiles visually with few data or performing no statistical test.

A previous study from our group reported a predictive model based on 16 geometrical descriptors that predicted PZAse kinetic parameters, explaining approximately 87% of the variability in k_cat_ [38]. Compared to our previous study and based on the recent availability of the WT-PZAse crystal structure [13], we considered Lys96 as part of the AS, and His57 as part of the MCS. Therefore, this study is a considerable improvement in our previous work, plus it includes stability and physicochemical aspects, besides the geometric descriptors. In contrast to previous studies based on a small number of mutants [42–46,74–76], our study includes a deeply statistical analysis based on 500 nanoseconds MD of every mutant PZAse. Therefore, discrepancies are expected to occur.

PZAse is a metalloenzyme that in-vitro could be activated by the coordination of several ions in the MCS [77,78]. Co, Cd, and Mn are the most important metal cofactors *in vitro*, although they may not have a significant effect *in vivo* given its low abundance in the intracellular environment. *In vivo*, previous studies found Fe [78] and Zn [77] to be coordinated with the PZAse in MTB. Therefore, it is likely that several metals may coordinate PZAse *in vivo*, with different abundances. Because of the lack of knowledge of this exact distribution of coordinated metals, plus the high computational demand for including the several 3D structural models for each metal coordinated, we decided as a first approach to only consider the apo-PZAse structure, in our calculations. We believe that the correct selection of metal coordinated PZAse and quantum mechanics calculations [79,80], may improve the prediction of the enzymatic function. For this, further studies are required.

There are several limitations during the development of a predictive model as the one described here. The experimental measurements of the kinetic parameters are highly variable between different batches; however, we controlled this by normalizing the parameters against the corresponding to the WT-PZAse. Also, available information about mutated PZAses and the measurement of its kinetic parameters is scarce compared with information related to the strain susceptibility. Also, we have focused our models on predicting enzymatic parameters of point mutations in PZAse without considering insertions or deletions, because the effects of this kind of mutations are not reliably predicted.

Based on this data, several studies pretended to dichotomous predict PZA susceptibility (resistant or susceptible) only from mutations in PZAse detected by *pncA* sequencing [81–83]. These approaches may be biased, with a risk of missing the correct physical/biological implications of PZAse mutations because PZA resistance could also be attributed to other factors besides PZAse activity itself, like differential PZAse expression levels [84] or dysfunction in other targets like *panD* [85], and many others still unknown [86]. This problem is explicit when a strain has PZAse mutations that do not affect the PZAse enzymatic function, but mutations in other critical genes [14,87,88] that generate PZA resistance in the bacteria. These cases would erroneously attribute PZAse mutations that do not affect the enzymatic function, the apparent effect of causing PZA resistance. This approach could be improved using whole-genome sequencing [89–92], but the expression and environmental factors are still present.

For these reasons, and considering that resistance to PZA can be attributed to multiple mechanisms, it is important to first predict PZAse enzymatic function from the *pncA* sequence. The only certain interpretation is to infer resistance to PZA when the *pncA* mutation causes a significant loss of enzymatic function. However, those mutations that do not significantly affect function, do not necessarily predict that the bacterium is susceptible, as mutations in genes associated with other resistance mechanisms may be present. In these cases, it is important to analyze mutations in other genes associated with alternative mechanisms of resistance to PZA.

In conclusion, the present work we show the construction of log-linear models based on geometrical, physicochemical, and stability descriptors derived from the modeled structure of the *M*. *tuberculosis* PZAse. This is useful to predict functional kinetic parameters of PZAse related to PZA resistance, based on the high certainty of PZAse dysfunction, from only the *pncA* gene sequence. This can be an important tool to contribute to efforts to detect early resistance to PZA.

## Supporting information

S1 Fig Ribbon representation of the structure of the *M*. *tuberculosis* PZAse protein. Highlighted in pink, residues from alpha helixes; in yellow, residues from beta-strands and in orange, residues from the flap region.
(TIF)

S2 Fig Tridimensional representations of geometrical reference points. (A) Points related to the active site (AS) and (B) points related to the metal coordination site (MCS). Vr represents the resultant vector of each residue.
(TIF)

S3 Fig DEPR profiles for WT and thirty-five mutated PZAses. Profiles of wild-type and mutated PZAses sorted by relative-k_cat_, each plot represent the difference in electrostatic potential respect to the wild-type for a given position.
(TIF)

S4 Fig NMA fluctuation profiles for WT and thirty-five mutated PZAses. Profiles of wild-type and mutated PZAses sorted by relative-k_cat_.
(TIF)

S5 Fig MD RMSD profiles for WT and thirty-five mutated PZAses. Profiles of wild-type and mutated PZAses sorted by relative-k_cat_, trajectories of 500 ns molecular dynamics of the entire protein were used as input.
(TIF)

S6 Fig MD RMSF profiles for WT and thirty-five mutated PZAses. Profiles of wild-type and mutated PZAses sorted by relative-k_cat_, trajectories of the last 100ns of a 500ns molecular dynamics of the protein backbone were used as input.
(TIF)

S7 Fig Per-position Boxplots for stability and physicochemical descriptors. In blue, PZAses with a k_cat_ greater or equal than 50. In red, PZAses with a k_cat_ lower than 50. (A, B) Fluctuations from NMA analysis. (C, D) RMSFs of the last 100ns from MD analysis (E, F) DEPRs from the MutantElec server.
(TIF)

S8 Fig Stability model (NMA) for k_cat_. (A) Table with estimated coefficients and statistics for the selected stability descriptors (fluctuations). (B) Comparison of statistics (R^2^, Adjusted R^2^, P-value, and RMSE) between the stability model and a random stability model. (C) Fitted values and experimental values for mean log_10_ (relative-k_cat_). (D) Heatmap showing the correlation coefficient between the selected descriptors. (E) Confidence intervals for the coefficients of each stability descriptor. (F) Distribution of RMSEs calculated by 6-fold cross-validation for the stability model (red) and a random model (blue).
(TIF)

S9 Fig Stability model (MD) for k_cat_. (A) Table with estimated coefficients and statistics for the selected stability descriptors (RMSFs). (B) Comparison of statistics (R^2^, Adjusted R^2^, P-value, and RMSE) between the stability model and a random stability model. (C) Fitted values and experimental values for mean log_10_ (relative-k_cat_). (D) Heatmap showing the correlation coefficient between the selected descriptors. (E) Confidence intervals for the coefficients of each stability descriptor. (F) Distribution of RMSEs calculated by 6-fold cross-validation for the stability model (red) and a random model (blue).
(TIF)

S10 Fig Physicochemical model for k_cat_. (A) Table with estimated coefficients and statistics for the selected physicochemical descriptors (DEPRs) (B) Comparison of statistics (R^2^, Adjusted R^2^, P-value, and RMSE) between the physicochemical model and a random physicochemical model. (C) Fitted values and experimental values for mean log_10_ (relative-k_cat_). (D) Heatmap showing the correlation coefficient between the selected descriptors. (E) Confidence intervals for the coefficients of each physicochemical descriptor. (F) Distribution of RMSEs calculated by 6-fold cross-validation for the physicochemical model (red) and a random model (blue).
(TIF)

S11 Fig Geometrical model for k_cat_. (A) Table with estimated coefficients and statistics for the selected geometrical descriptors (B) Comparison of statistics (R^2^, Adjusted R^2^, P-value, and RMSE) between the geometrical model and a random geometrical model. (C) Fitted values and experimental values for mean log_10_ (relative-k_cat_). (D) Heatmap showing the correlation coefficient between the selected descriptors. (E) Confidence intervals for the coefficients of each geometrical descriptor. (F) Distribution of RMSEs calculated by 6-fold cross-validation for the geometrical model (red) and a random model (blue).
(TIF)

S12 Fig Stability model (NMA) for K_M_. (A) Table with estimated coefficients and statistics for the selected stability descriptors (fluctuations), (B) Comparison of statistics (R^2^, Adjusted R^2^, P-value, and RMSE) between the stability model and a random stability model. (C) Fitted values and experimental values for mean log_10_ (relative-K_M_). (D) Heatmap showing the correlation coefficient between the selected descriptors. (E) Confidence intervals for the coefficients of each stability descriptor. (F) Distribution of RMSEs calculated by 6-fold cross-validation for the stability model (red) and a random model (blue).
(TIF)

S13 Fig Stability model (MD) for K_M_. (A) Table with estimated coefficients and statistics for the selected stability descriptors (RMSFs). (B) Comparison of statistics (R^2^, Adjusted R^2^, P-value, and RMSE) between the stability model and a random stability model. (C) Fitted values and experimental values for mean log_10_ (relative-K_M_). (D) Heatmap showing the correlation coefficient between the selected descriptors. (E) Confidence intervals for the coefficients of each stability descriptor. (F) Distribution of RMSEs calculated by 6-fold cross-validation for the stability model (red) and a random model (blue).
(TIF)

S14 Fig Physicochemical model for K_M_. (A) Table with estimated coefficients and statistics for the selected physicochemical descriptors (DEPRs). (B) Comparison of statistics (R^2^, Adjusted R^2^, P-value, and RMSE) between the physicochemical model and a random physicochemical model. (C) Fitted values and experimental values for mean log_10_ (relative-K_M_). (D) Heatmap showing the correlation coefficient between the selected descriptors. (E) Confidence intervals for the coefficients of each physicochemical descriptor. (F) Distribution of RMSEs calculated by 6-fold cross-validation for the physicochemical model (red) and a random model (blue).
(TIF)

S15 Fig Geometrical model for K_M_. (A) Table with estimated coefficients and statistics for the selected geometrical descriptors. (B) Comparison of statistics (R^2^, Adjusted R^2^, P-value, and RMSE) between the geometrical model and a random geometrical model. (C) Fitted values and experimental values for mean log_10_ (relative-K_M_). (D) Heatmap showing the correlation coefficient between the selected descriptors. (E) Confidence intervals for the coefficients of each geometrical descriptor. (F) Distribution of RMSEs calculated by 6-fold cross-validation for the geometrical model (red) and a random model (blue).
(TIF)

S16 Fig Stability model (NMA) for efficiency. (A) Table with estimated coefficients and statistics for the selected stability descriptors (fluctuations). (B) Comparison of statistics (R^2^, Adjusted R^2^, P-value, and RMSE) between the stability model and a random stability model. (C) Fitted values and experimental values for mean log_10_ (relative efficiency). (D) Heatmap showing the correlation coefficient between the selected descriptors. (E) Confidence intervals for the coefficients of each stability descriptor. (F) Distribution of RMSEs calculated by 6-fold cross-validation for the stability model (red) and a random model (blue).
(TIF)

S17 Fig Stability model (MD) for efficiency. (A) Table with estimated coefficients and statistics for the selected stability descriptors (RMSFs). (B) Comparison of statistics (R^2^, Adjusted R^2^, P-value, and RMSE) between the stability model and a random stability model. (C) Fitted values and experimental values for mean log_10_ (relative efficiency). (D) Heatmap showing the correlation coefficient between the selected descriptors. (E) Confidence intervals for the coefficients of each stability descriptor. (F) Distribution of RMSEs calculated by 6-fold cross-validation for the stability model (red) and a random model (blue).
(TIF)

S18 Fig Physicochemical model for efficiency. (A) Table with estimated coefficients and statistics for the selected physicochemical descriptors (DEPRs). (B) Comparison of statistics (R^2^, Adjusted R^2^, P-value, and RMSE) between the physicochemical model and a random physicochemical model. (C) Fitted values and experimental values for mean log_10_ (relative efficiency). (D) Heatmap showing the correlation coefficient between the selected descriptors. (E) Confidence intervals for the coefficients of each physicochemical descriptor. (F) Distribution of RMSEs calculated by 6-fold cross-validation for the physicochemical model (red) and a random model (blue).
(TIF)

S19 Fig Geometrical model for efficiency. (A) Table with estimated coefficients and statistics for the selected geometrical descriptors. (B) Comparison of statistics (R^2^, Adjusted R^2^, P-value, and RMSE) between the geometrical model and a random geometrical model. (C) Fitted values and experimental values for mean log_10_ (relative efficiency). (D) Heatmap showing the correlation coefficient between the selected descriptors. (E) Confidence intervals for the coefficients of each geometrical descriptor. (F) Distribution of RMSEs calculated by 6-fold cross-validation for the geometrical model (red) and a random model (blue).
(TIF)

S20 Fig Stability model (NMA) for activity. (A) Table with estimated coefficients and statistics for the selected stability descriptors (fluctuations). (B) Comparison of statistics (R^2^, Adjusted R^2^, P-value, and RMSE) between the stability model and a random stability model. (C) Fitted values and experimental values for mean log_10_ (relative activity). (D) Heatmap showing the correlation coefficient between the selected descriptors. (E) Confidence intervals for the coefficients of each stability descriptor. (F) Distribution of RMSEs calculated by 6-fold cross-validation for the stability model (red) and a random model (blue).
(TIF)

S21 Fig Stability model (MD) for activity. (A) Table with estimated coefficients and statistics for the selected stability descriptors (RMSFs). (B) Comparison of statistics (R^2^, Adjusted R^2^, P-value, and RMSE) between the stability model and a random stability model. (C) Fitted values and experimental values for mean log_10_ (relative activity). (D) Heatmap showing the correlation coefficient between the selected descriptors. (E) Confidence intervals for the coefficients of each stability descriptor. (F) Distribution of RMSEs calculated by 6-fold cross-validation for the stability model (red) and a random model (blue).
(TIF)

S22 Fig Physicochemical model for activity. (A) Table with estimated coefficients and statistics for the selected physicochemical descriptors (DEPRs). (B) Comparison of statistics (R^2^, Adjusted R^2^, P-value, and RMSE) between the physicochemical model and a random physicochemical model. (C) Fitted values and experimental values for mean log_10_ (relative activity). (D) Heatmap showing the correlation coefficient between the selected descriptors. (E) Confidence intervals for the coefficients of each physicochemical descriptor. (F) Distribution of RMSEs calculated by 6-fold cross-validation for the physicochemical model (red) and a random model (blue).
(TIF)

S23 Fig Geometrical model for activity. (A) Table with estimated coefficients and statistics for the selected geometrical descriptors. (B) Comparison of statistics (R^2^, Adjusted R^2^, P-value00, and RMSE) between the geometrical model and a random geometrical model. (C) Fitted values and experimental values for mean log_10_ (relative activity). (D) Heatmap showing the correlation coefficient between the selected descriptors. (E) Confidence intervals for the coefficients of each geometrical descriptor. (F) Distribution of RMSEs calculated by 6-fold cross-validation for the geometrical model (red) and a random model (blue).
(TIF)

S24 Fig Weighted model (NMA) for k_cat_. (A) Table with estimated coefficients and statistics for the individual predictions of stability (NMA), physicochemical and geometrical models. (B) Comparison among the individual models and the weighted model (NMA) for k_cat_. (C) Fitted values and experimental values for mean log_10_ (relative-k_cat_) for the weighted model (NMA). (D) Distribution of RMSE calculated by 6-fold cross-validation for the weighted and individual models.
(TIF)

S25 Fig Weighted model (MD) for k_cat_. (A) Table with estimated coefficients and statistics for the individual predictions of stability (MD), physicochemical and geometrical models. (B) Comparison among the individual models and the weighted model (MD) for k_cat_. (C) Fitted values and experimental values for mean log_10_ (relative-k_cat_) for the weighted model (MD). (D) Distribution of RMSE calculated by 6-fold cross-validation for the weighted and individual models.
(TIF)

S26 Fig Weighted model (NMA) for K_M_. (A) Table with estimated coefficients and statistics for the individual predictions of stability (NMA), physicochemical and geometrical models. (B) Comparison among the individual models and the weighted model (NMA) for K_M_. (C) Fitted values and experimental values for mean log_10_ (relative- K_M_) for the weighted model (NMA). (D) Distribution of RMSE calculated by 6-fold cross-validation for the weighted and individual models.
(TIF)

S27 Fig Weighted model (MD) for K_M_. (A) Table with estimated coefficients and statistics for the individual predictions of stability (MD), physicochemical and geometrical models. (B) Comparison among the individual models and the weighted model (MD) for K_M_. (C) Fitted values and experimental values for mean log_10_ (relative-K_M_) for the weighted model (MD). (D) Distribution of RMSE calculated by 6-fold cross-validation for the weighted and individual models.
(TIF)

S28 Fig Weighted model (NMA) for efficiency. (A) Table with estimated coefficients and statistics for the individual predictions of stability (NMA), physicochemical and geometrical models. (B) Comparison among the individual models and the weighted model (NMA) for efficiency. (C) Fitted values and experimental values for mean log_10_ (relative efficiency) for the weighted model (NMA). (D) Distribution of RMSE calculated by 6-fold cross-validation for the weighted and individual models.
(TIF)

S29 Fig Weighted model (MD) for efficiency. (A) Table with estimated coefficients and statistics for the individual predictions of stability (MD), physicochemical and geometrical models. (B) Comparison among the individual models and the weighted model (MD) for efficiency. (C) Fitted values and experimental values for mean log_10_ (relative efficiency) for the weighted model (MD). (D) Distribution of RMSE calculated by 6-fold cross-validation for the weighted and individual models.
(TIF)

S30 Fig Weighted model (NMA) for activity. (A) Table with estimated coefficients and statistics for the individual predictions of stability (NMA), physicochemical and geometrical models. (B) Comparison among the individual models and the weighted model (NMA) for activity. (C) Fitted values and experimental values for mean log_10_ (relative activity) for the weighted model (NMA). (D) Distribution of RMSE calculated by 6-fold cross-validation for the weighted and individual models.
(TIF)

S31 Fig Weighted model (MD) for activity. (A) Table with estimated coefficients and statistics for the individual predictions of stability (MD), physicochemical and geometrical models. (B) Comparison among the individual models and the weighted model (MD) for activity. (C) Fitted values and experimental values for mean log_10_ (relative activity) for the weighted model (MD). (D) Distribution of RMSE calculated by 6-fold cross-validation for the weighted and individual models.
(TIF)

S1 Dataset Dataset of geometrical descriptors per residue of WT-PZAse and 35 mutants. In gray, mutants from the second batch.
(XLSX)

S2 Dataset Dataset of physicochemical descriptors per residue of WT-PZAse and 35 mutants. In gray, mutants from the second batch.
(XLSX)

S3 Dataset Dataset of fluctuations per residue from NMA analysis of WT-PZAse and 35 mutants. In gray, mutants from the second batch.
(XLSX)

S4 Dataset Dataset of fluctuations per residue from MD analysis of WT-PZAse and 35 mutants. In gray, mutants from the second batch.
(XLSX)

S1 Table Absolute and relative standard errors for kinetic parameters of two batches of mutated PZAses from *M*. *tuberculosis*. (XLSX)

S2 Table The distance matrix for selected residues for each model to the residues of AS and MCS. The descriptors that were selected in more than one model are in bold.
(XLSX)

S1 Appendix List of geometrical descriptors used in geometrical modeling. A generalized meaning of each of them.
(XLSX)
