# Supplementary figures and images for: Prediction of Mycobacterium tuberculosis pyrazinamidase function based on structural stability, physicochemical and geometrical descriptors
Source: PLoS One. 2020 Jul 31;15(7):e0235643. doi: 10.1371/journal.pone.0235643 (PMC7394417; doi:10.1371/journal.pone.0235643)

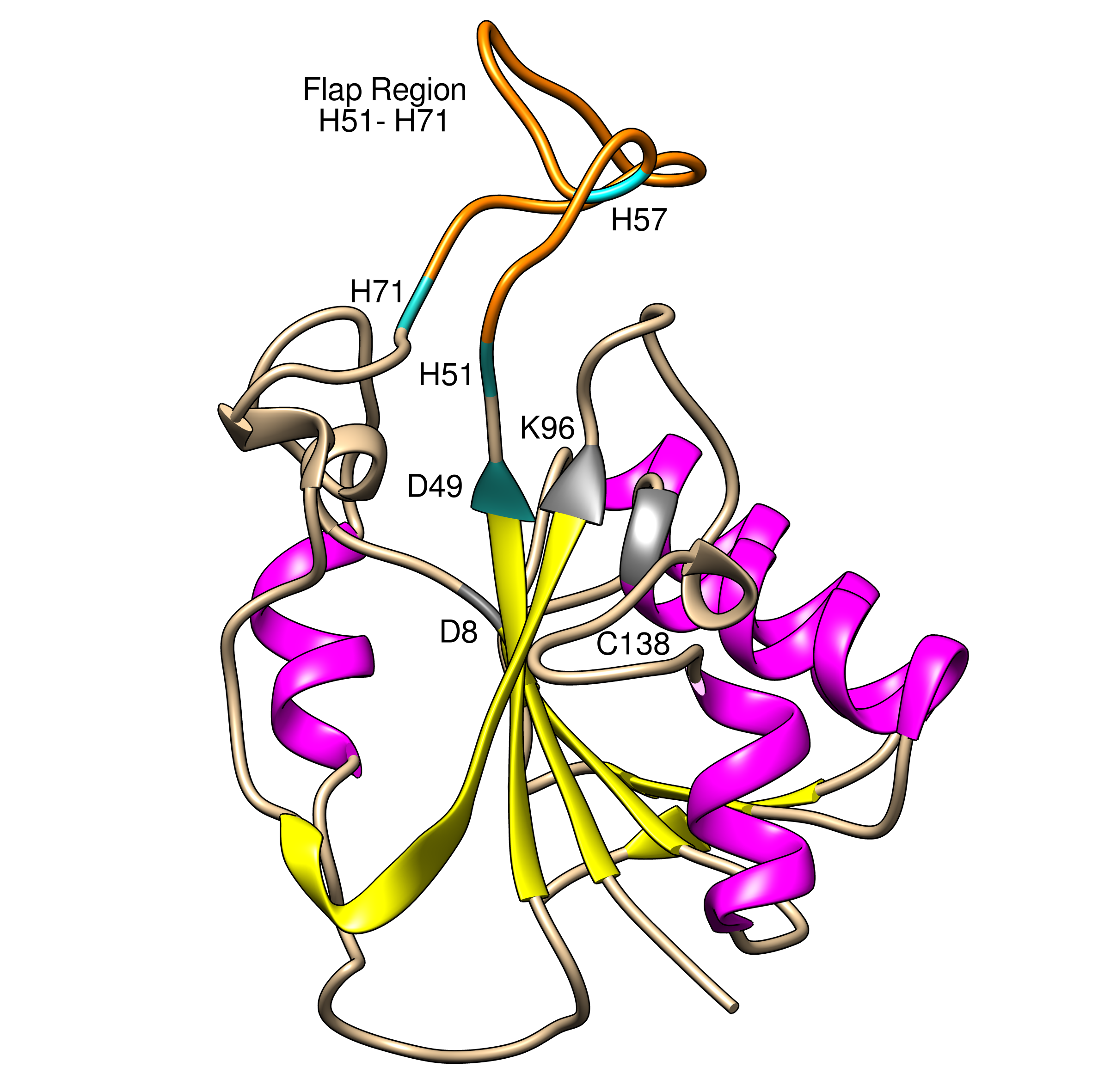

Supplement: S1 Fig — Highlighted in pink, residues from alpha helixes; in yellow, residues from beta-strands and in orange, residues from the flap region. (TIF) [file pone.0235643.s001.tif]

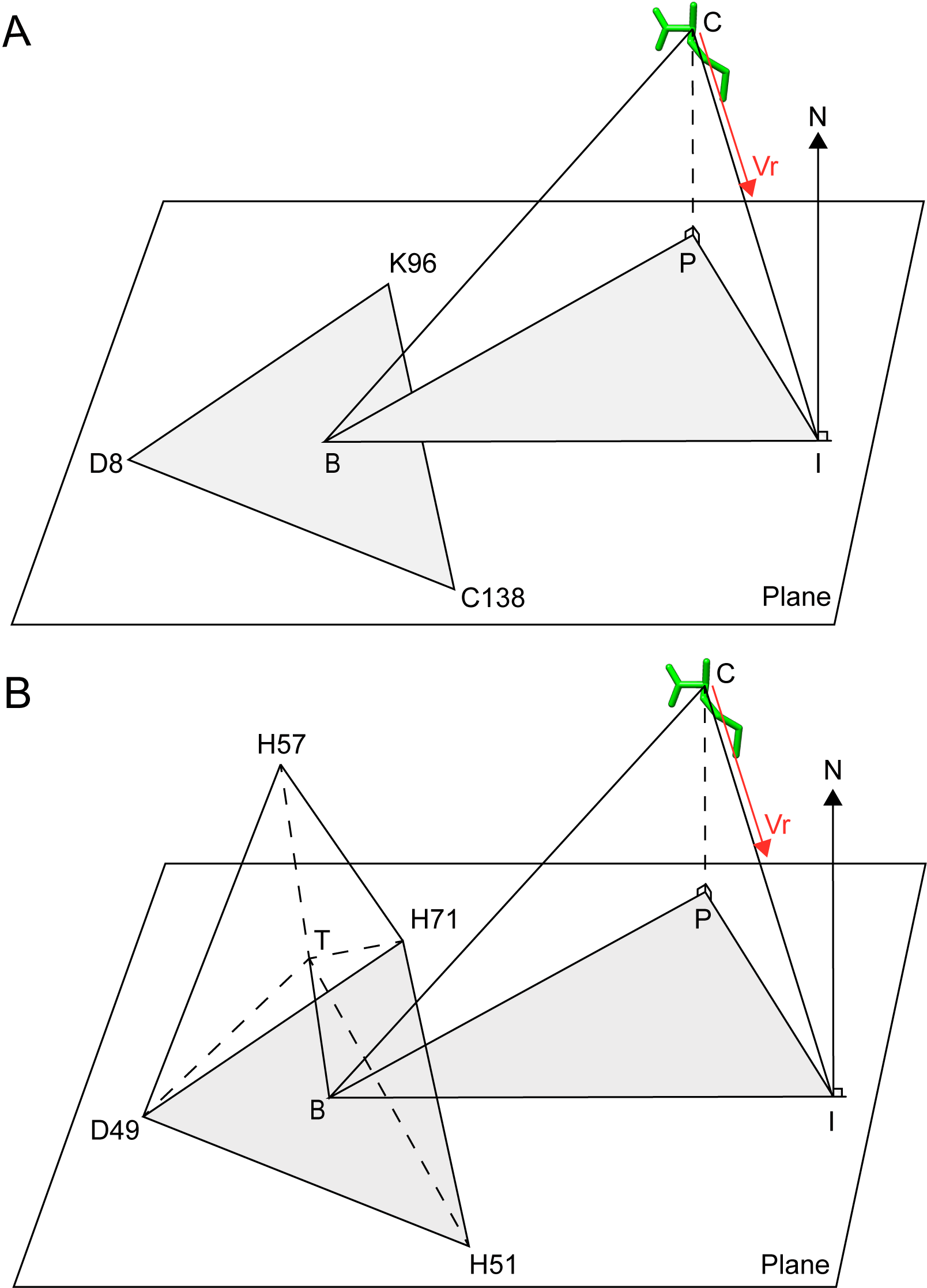

Supplement: S2 Fig — (A) Points related to the active site (AS) and (B) points related to the metal coordination site (MCS). Vr represents the resultant vector of each residue. (TIF) [file pone.0235643.s002.tif]

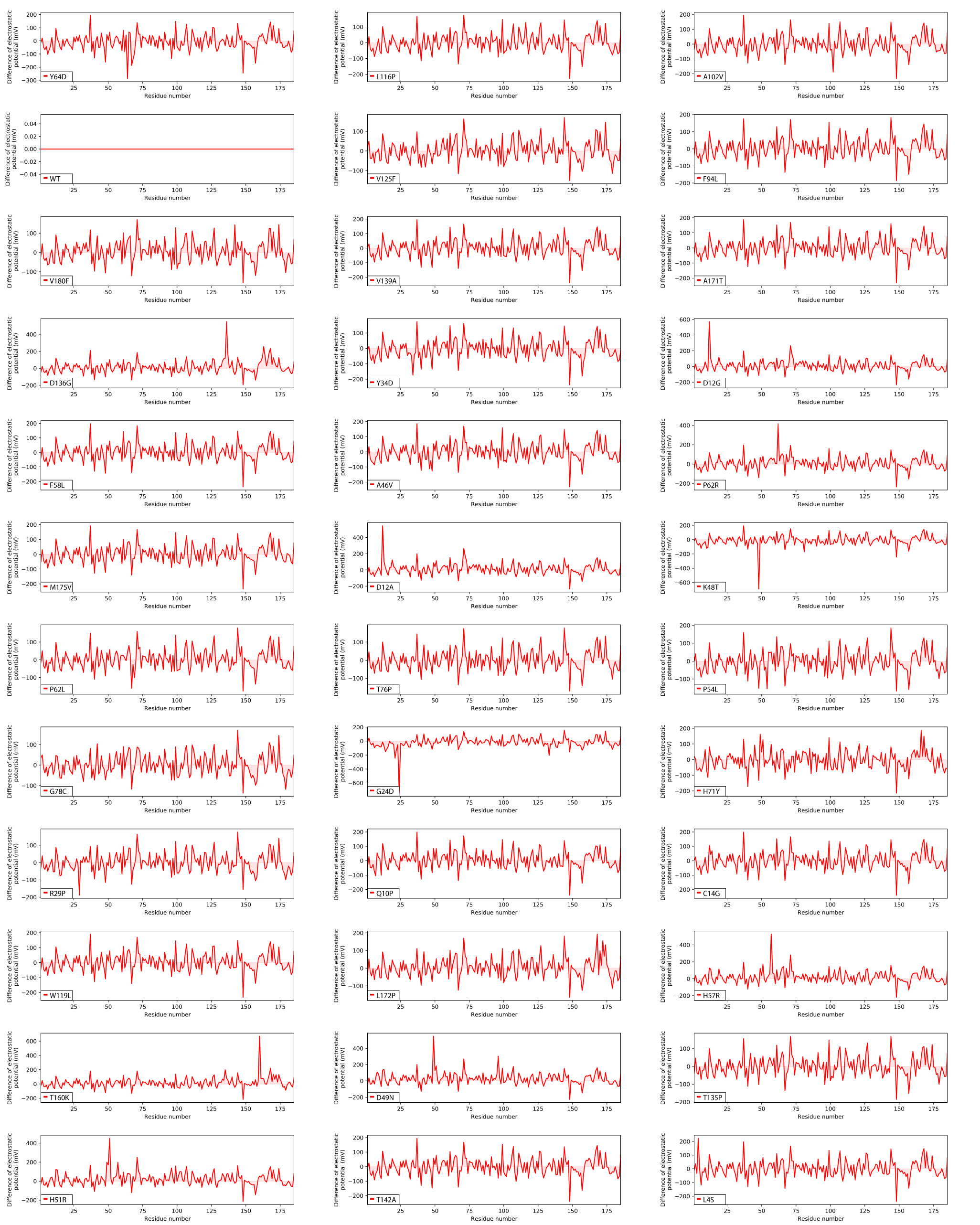

Supplement: S3 Fig — Profiles of wild-type and mutated PZAses sorted by relative-kcat, each plot represent the difference in electrostatic potential respect to the wild-type for a given position. (TIF) [file pone.0235643.s003.tif]

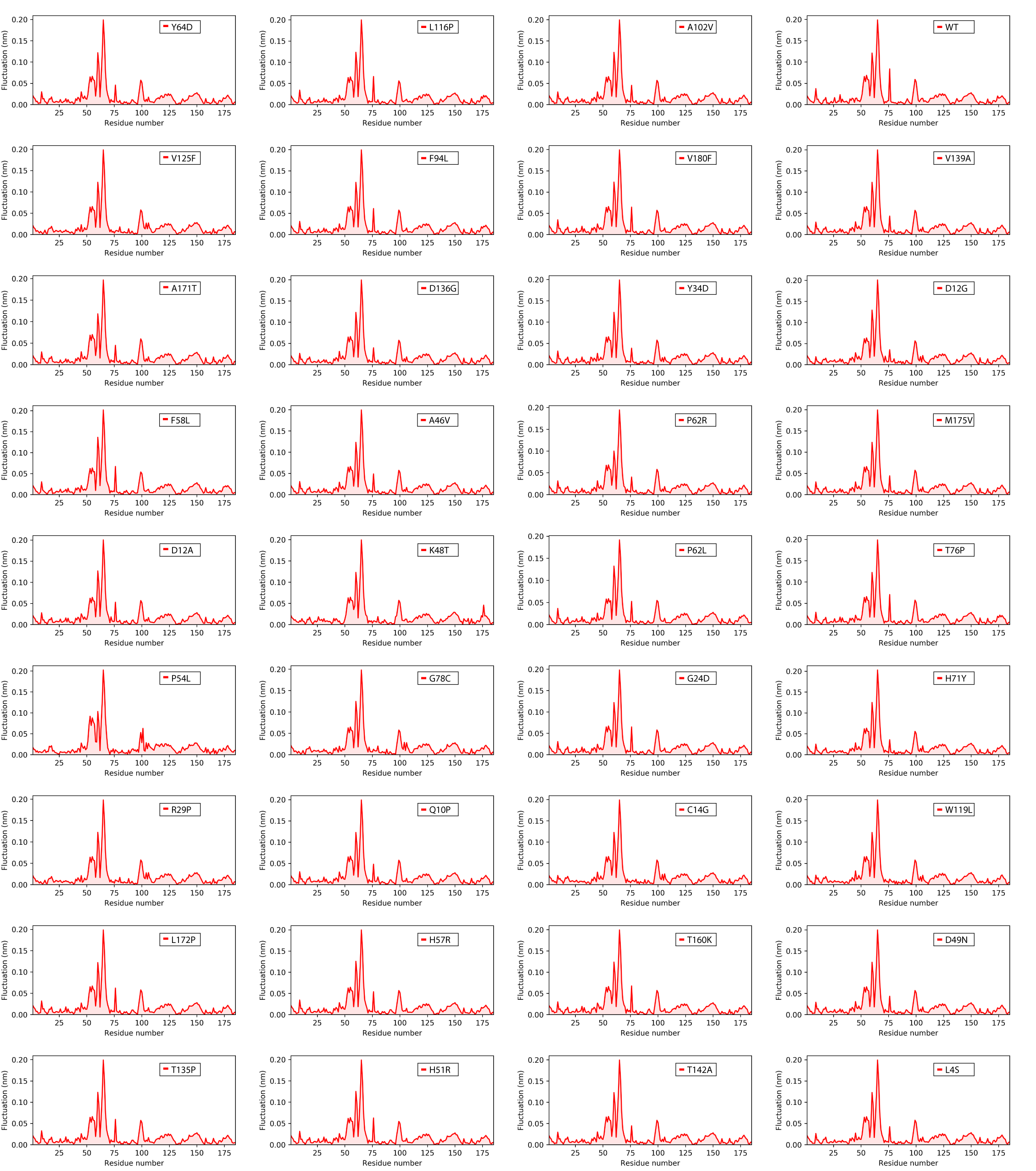

Supplement: S4 Fig — Profiles of wild-type and mutated PZAses sorted by relative-kcat. (TIF) [file pone.0235643.s004.tif]

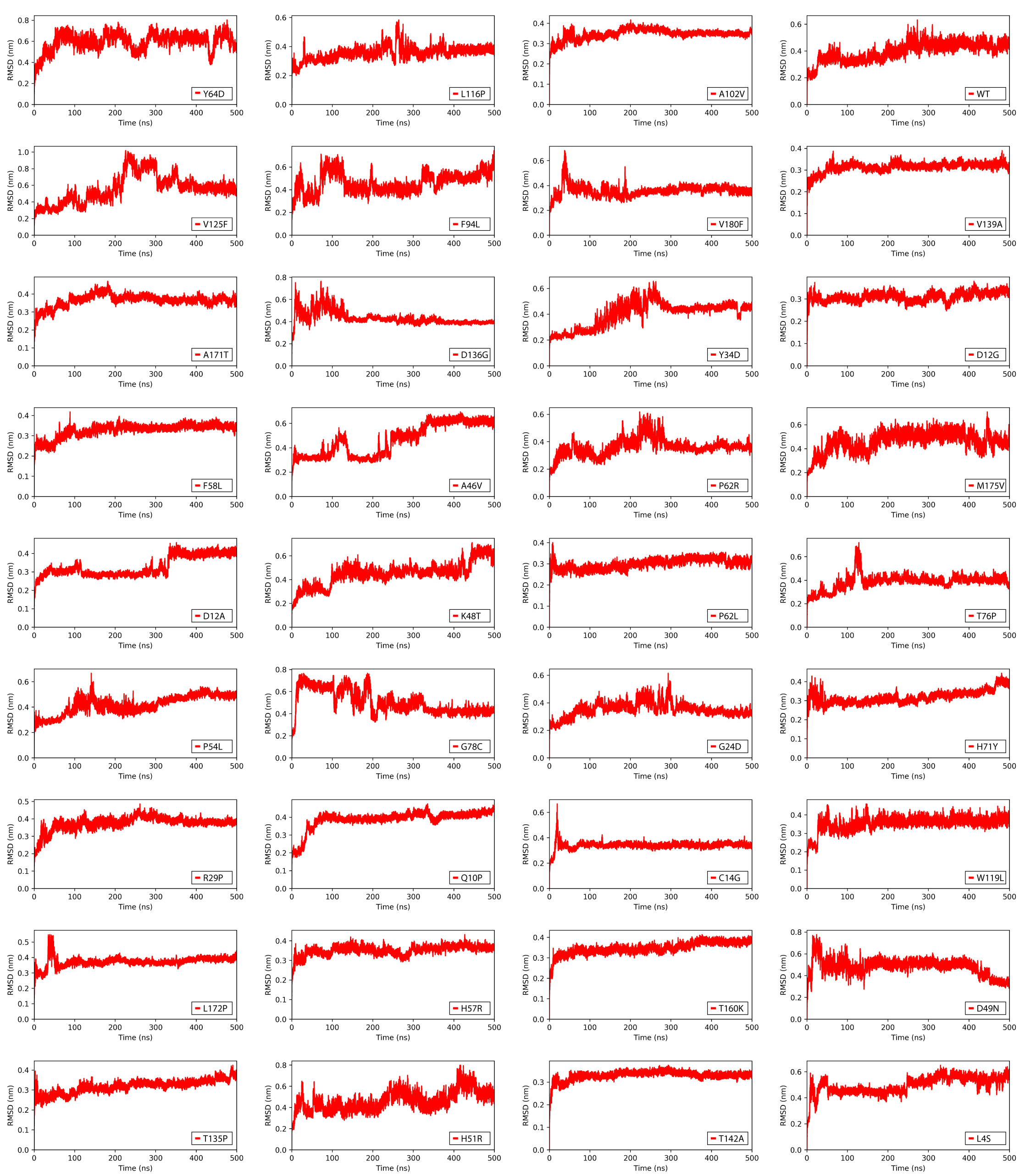

Supplement: S5 Fig — Profiles of wild-type and mutated PZAses sorted by relative-kcat, trajectories of 500 ns molecular dynamics of the entire protein were used as input. (TIF) [file pone.0235643.s005.tif]

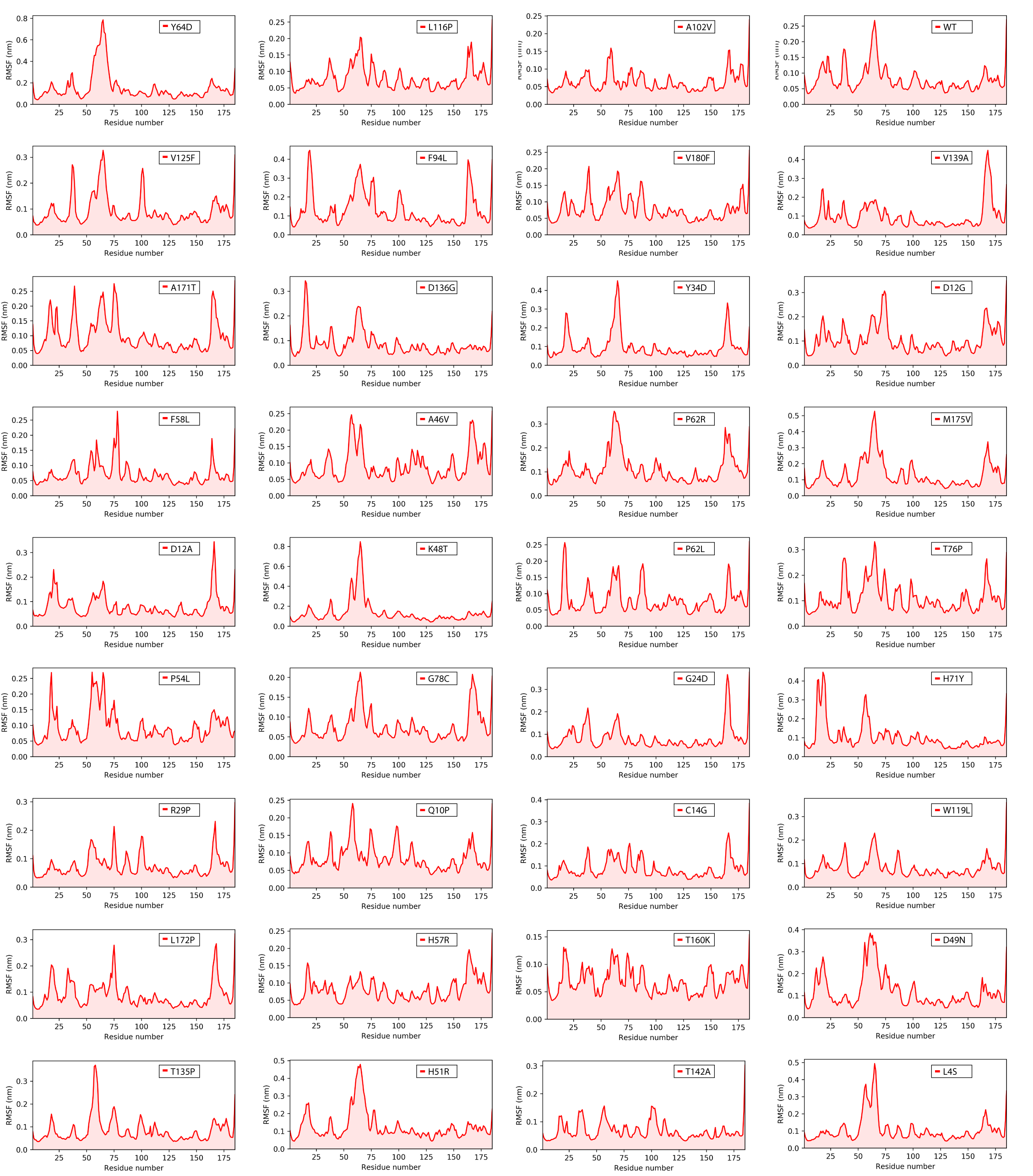

Supplement: S6 Fig — Profiles of wild-type and mutated PZAses sorted by relative-kcat, trajectories of the last 100ns of a 500ns molecular dynamics of the protein backbone were used as input. (TIF) [file pone.0235643.s006.tif]

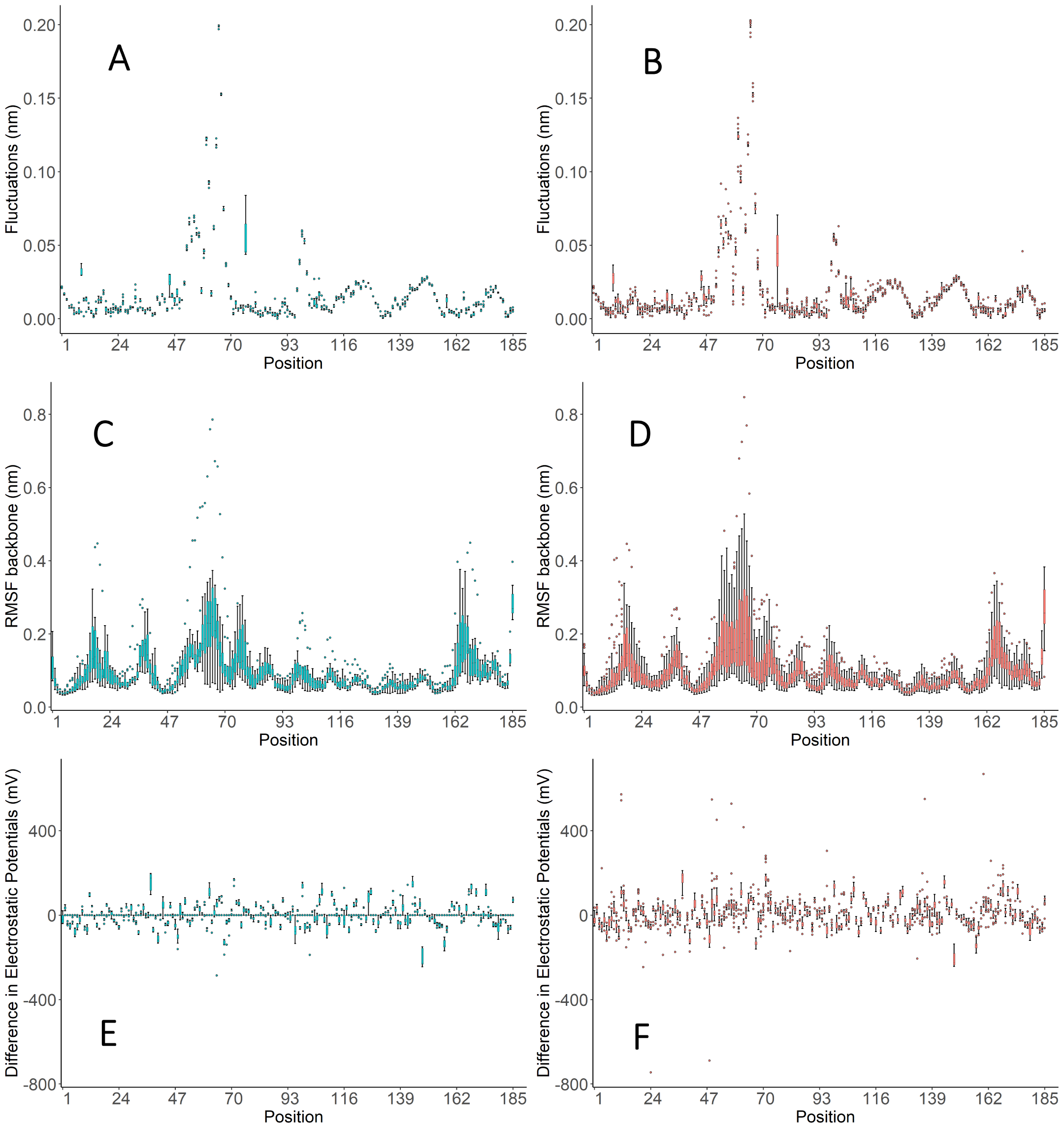

Supplement: S7 Fig — In blue, PZAses with a kcat greater or equal than 50. In red, PZAses with a kcat lower than 50. (A, B) Fluctuations from NMA analysis. (C, D) RMSFs of the last 100ns from MD analysis (E, F) DEPRs from the MutantElec server. (TIF) [file pone.0235643.s007.tif]

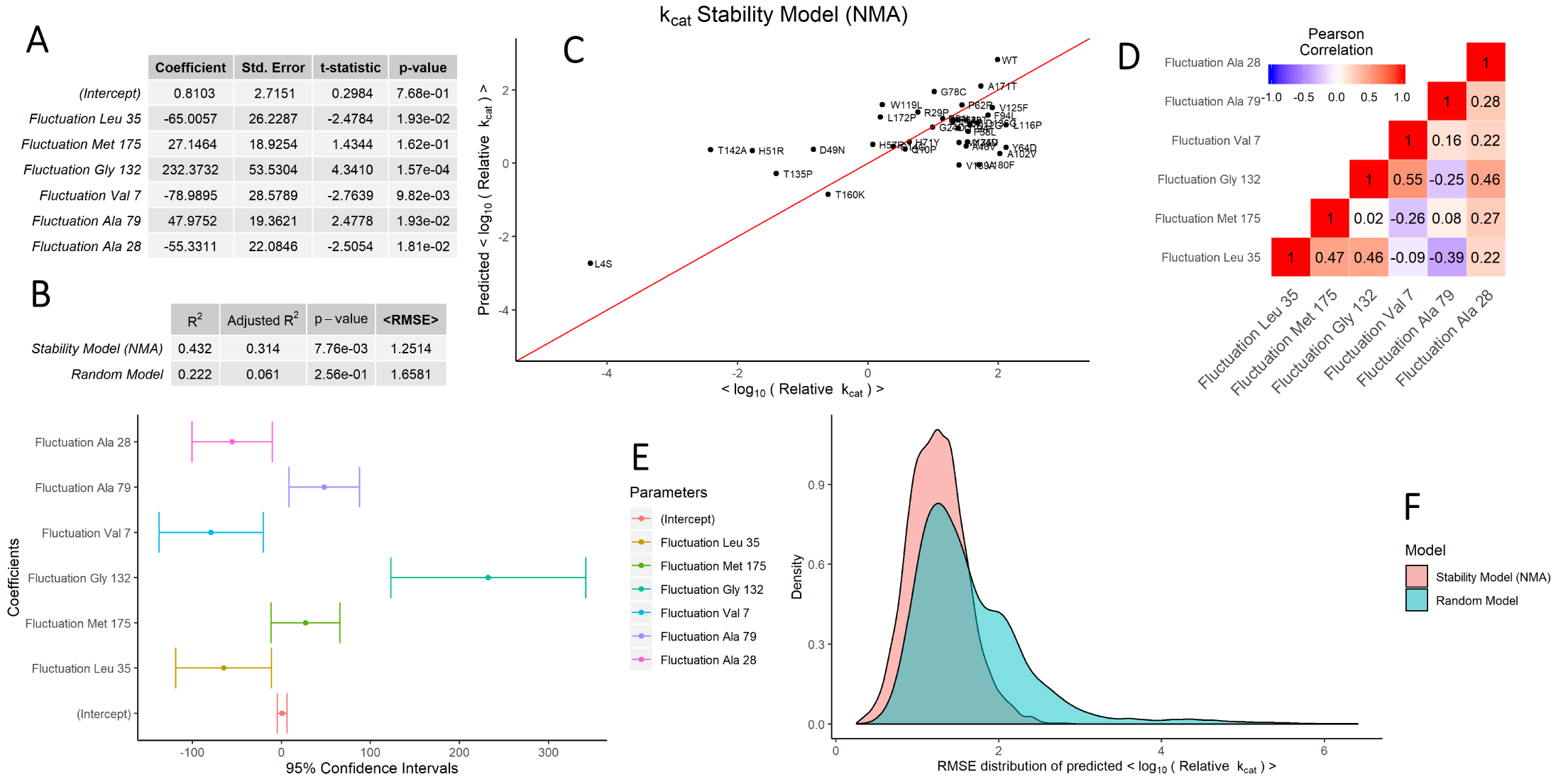

Supplement: S8 Fig — (A) Table with estimated coefficients and statistics for the selected stability descriptors (fluctuations). (B) Comparison of statistics (R2, Adjusted R2, P-value, and RMSE) between the stability model and a random stability model. (C) Fitted values and experimental values for mean log10 (relative-kcat). (D) Heatmap showing the correlation coefficient between the selected descriptors. (E) Confidence intervals for the coefficients of each stability descriptor. (F) Distribution of RMSEs calculated by 6-fold cross-validation for the stability model (red) and a random model (blue). (TIF) [file pone.0235643.s008.tif]

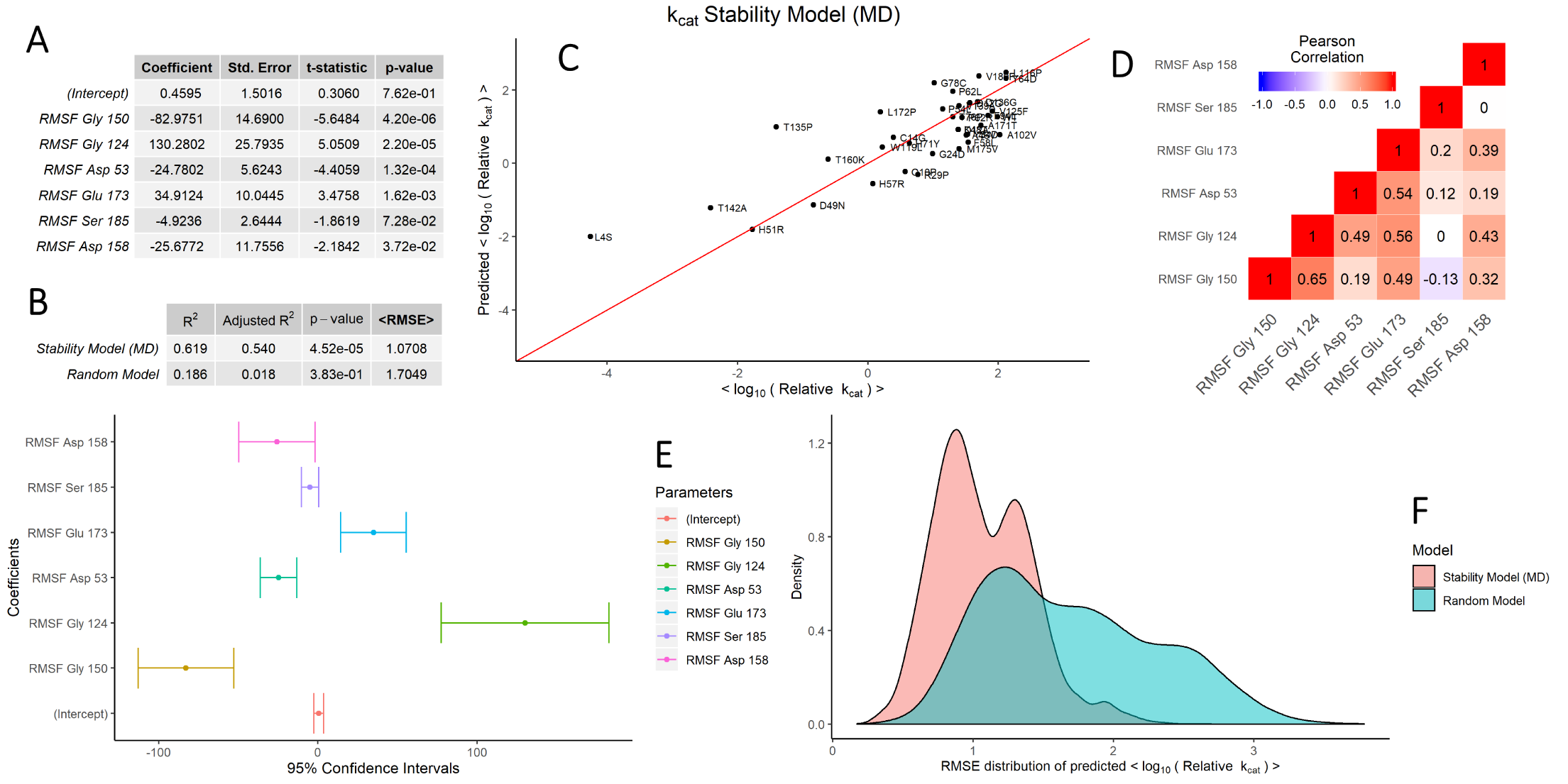

Supplement: S9 Fig — (A) Table with estimated coefficients and statistics for the selected stability descriptors (RMSFs). (B) Comparison of statistics (R2, Adjusted R2, P-value, and RMSE) between the stability model and a random stability model. (C) Fitted values and experimental values for mean log10 (relative-kcat). (D) Heatmap showing the correlation coefficient between the selected descriptors. (E) Confidence intervals for the coefficients of each stability descriptor. (F) Distribution of RMSEs calculated by 6-fold cross-validation for the stability model (red) and a random model (blue). (TIF) [file pone.0235643.s009.tif]

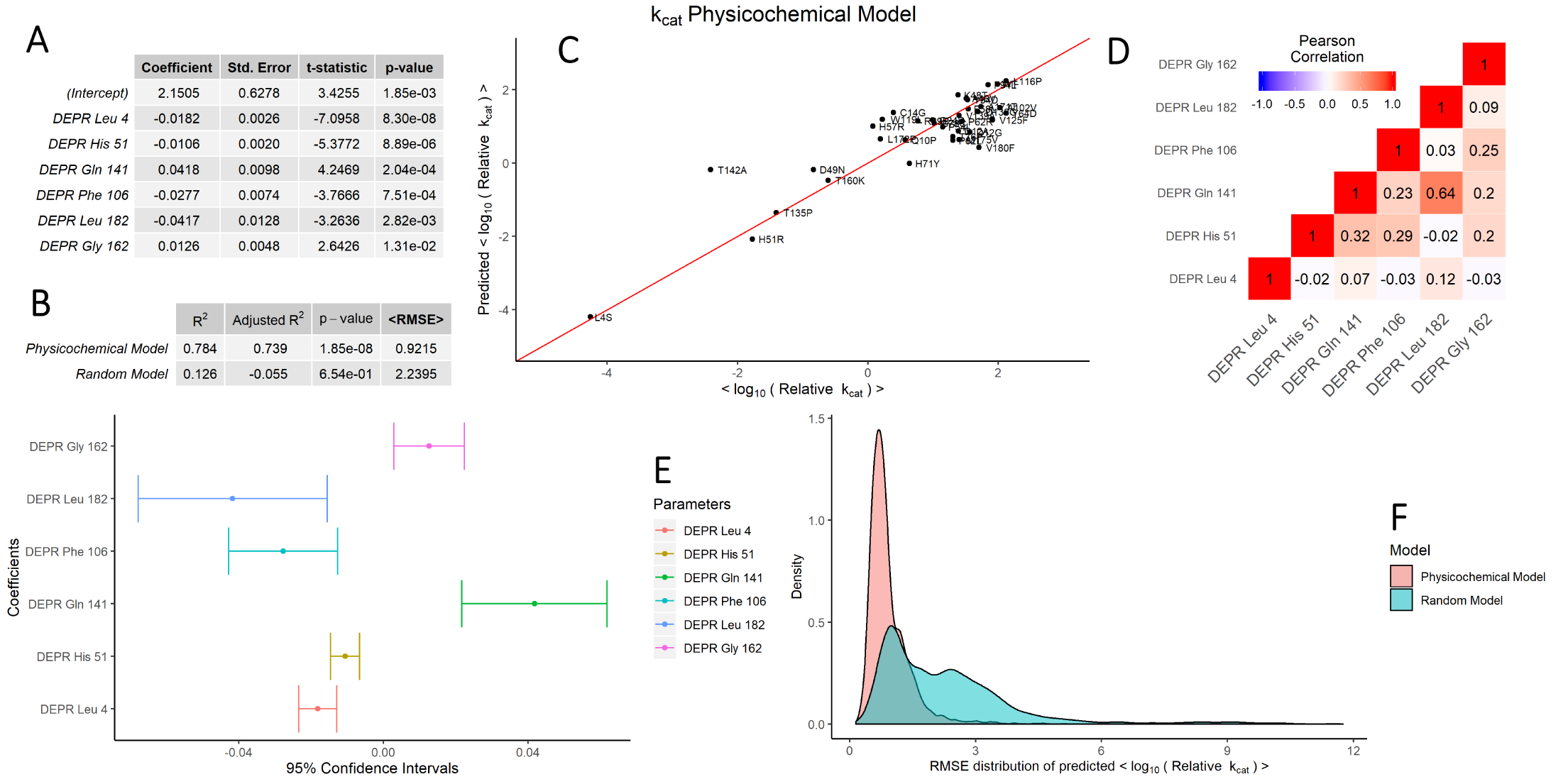

Supplement: S10 Fig — (A) Table with estimated coefficients and statistics for the selected physicochemical descriptors (DEPRs) (B) Comparison of statistics (R2, Adjusted R2, P-value, and RMSE) between the physicochemical model and a random physicochemical model. (C) Fitted values and experimental values for mean log10 (relative-kcat). (D) Heatmap showing the correlation coefficient between the selected descriptors. (E) Confidence intervals for the coefficients of each physicochemical descriptor. (F) Distribution of RMSEs calculated by 6-fold cross-validation for the physicochemical model (red) and a random model (blue). (TIF) [file pone.0235643.s010.tif]

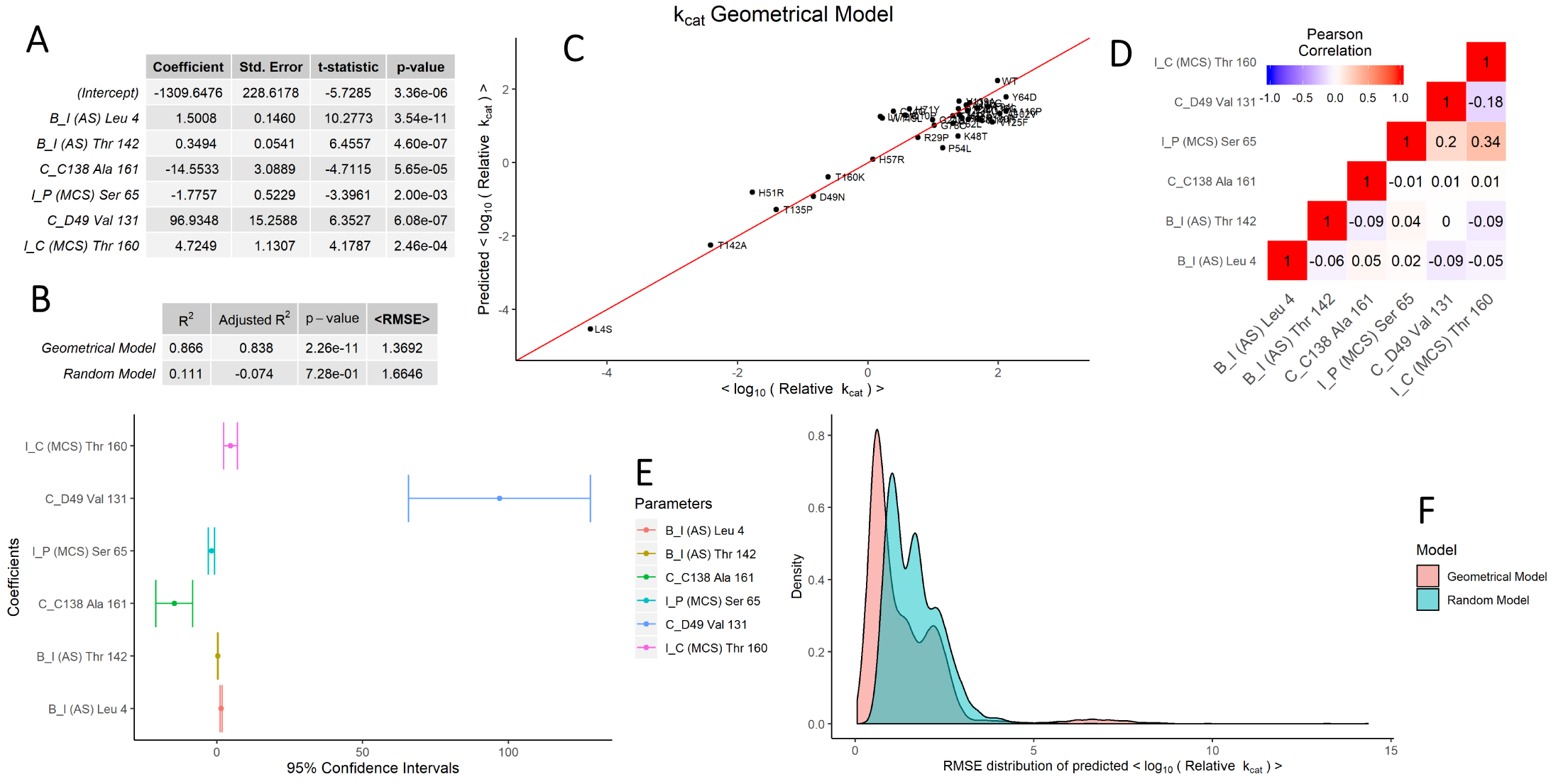

Supplement: S11 Fig — (A) Table with estimated coefficients and statistics for the selected geometrical descriptors (B) Comparison of statistics (R2, Adjusted R2, P-value, and RMSE) between the geometrical model and a random geometrical model. (C) Fitted values and experimental values for mean log10 (relative-kcat). (D) Heatmap showing the correlation coefficient between the selected descriptors. (E) Confidence intervals for the coefficients of each geometrical descriptor. (F) Distribution of RMSEs calculated by 6-fold cross-validation for the geometrical model (red) and a random model (blue). (TIF) [file pone.0235643.s011.tif]

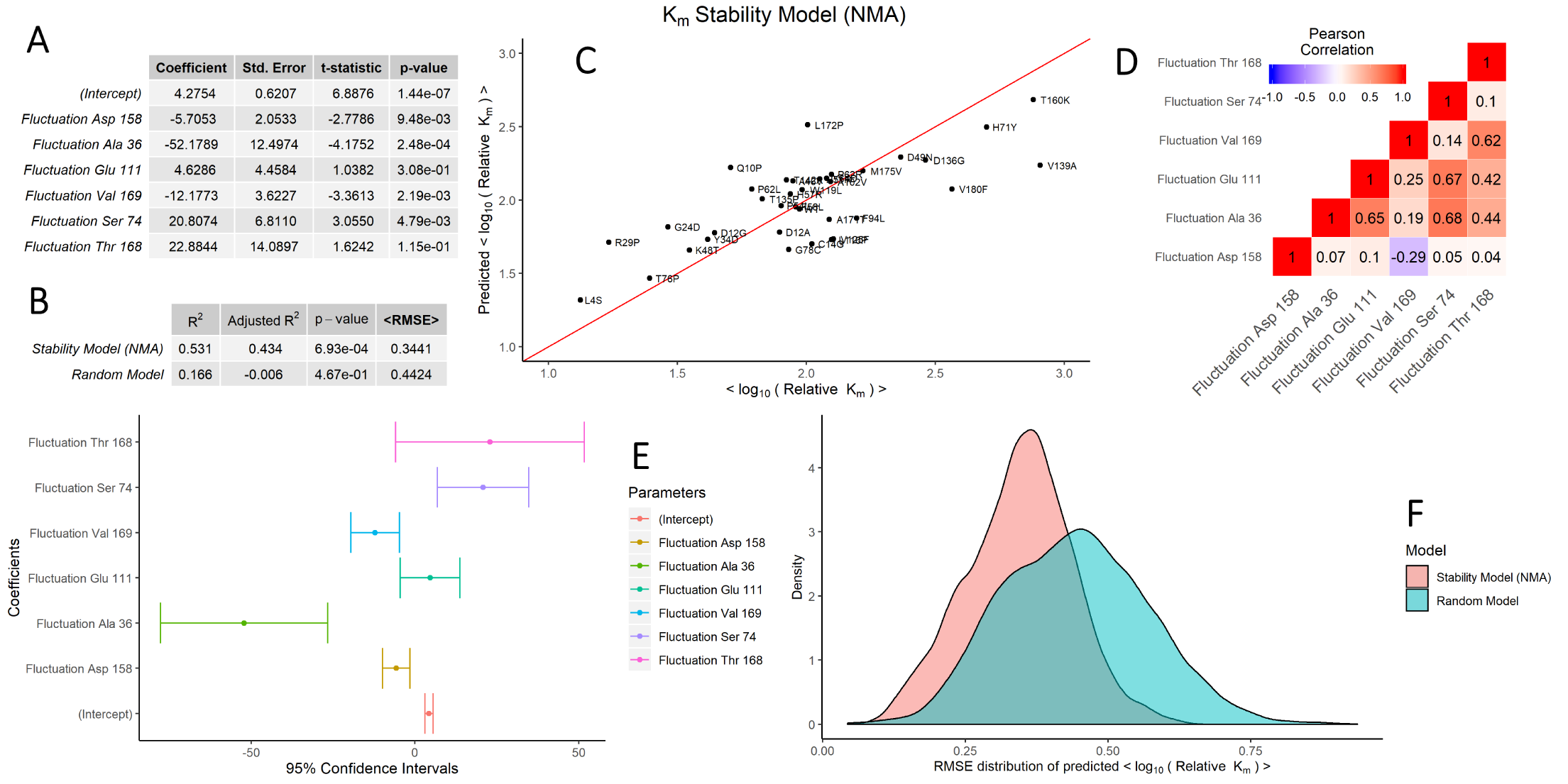

Supplement: S12 Fig — (A) Table with estimated coefficients and statistics for the selected stability descriptors (fluctuations), (B) Comparison of statistics (R2, Adjusted R2, P-value, and RMSE) between the stability model and a random stability model. (C) Fitted values and experimental values for mean log10 (relative-KM). (D) Heatmap showing the correlation coefficient between the selected descriptors. (E) Confidence intervals for the coefficients of each stability descriptor. (F) Distribution of RMSEs calculated by 6-fold cross-validation for the stability model (red) and a random model (blue). (TIF) [file pone.0235643.s012.tif]

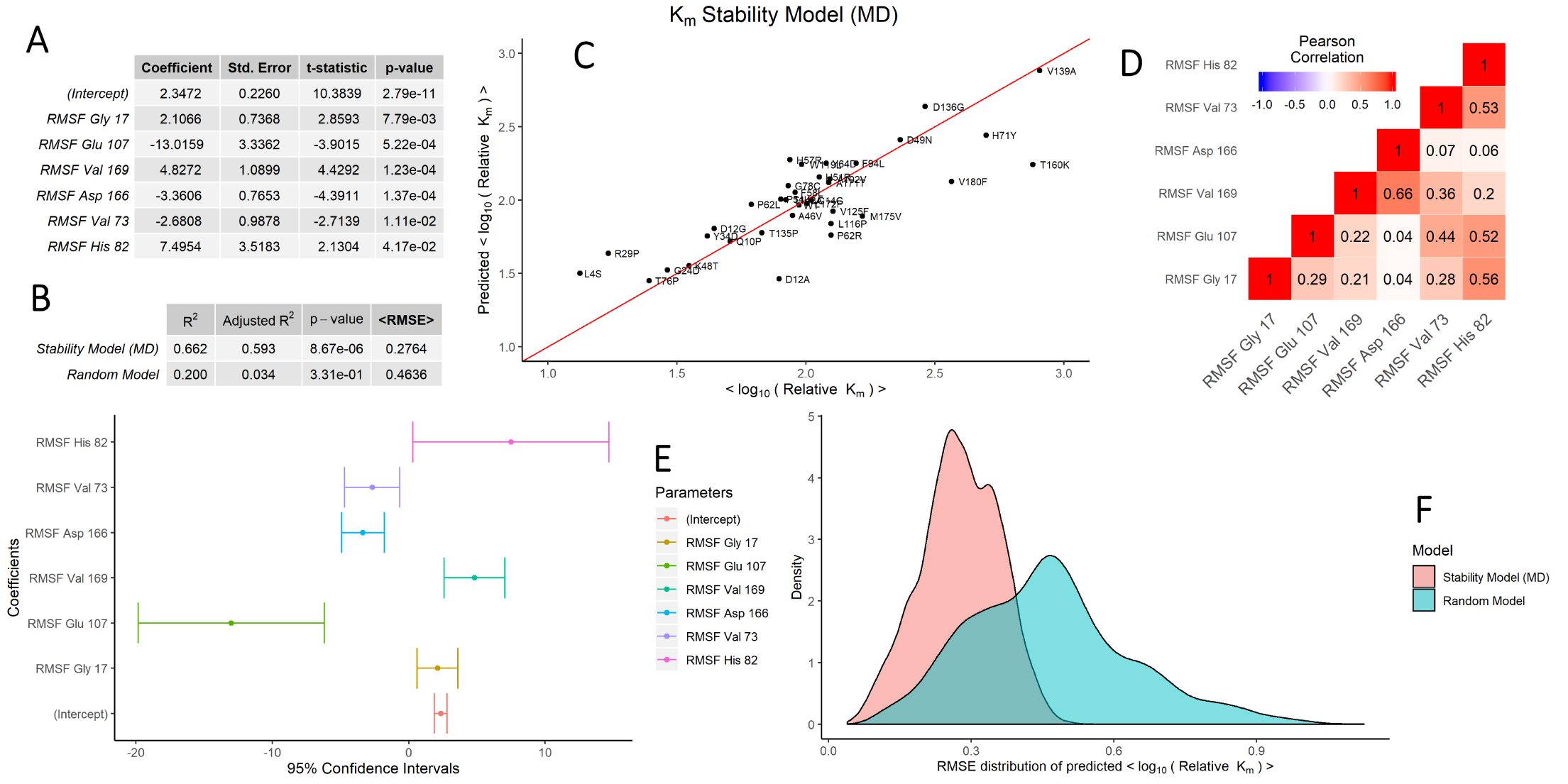

Supplement: S13 Fig — (A) Table with estimated coefficients and statistics for the selected stability descriptors (RMSFs). (B) Comparison of statistics (R2, Adjusted R2, P-value, and RMSE) between the stability model and a random stability model. (C) Fitted values and experimental values for mean log10 (relative-KM). (D) Heatmap showing the correlation coefficient between the selected descriptors. (E) Confidence intervals for the coefficients of each stability descriptor. (F) Distribution of RMSEs calculated by 6-fold cross-validation for the stability model (red) and a random model (blue). (TIF) [file pone.0235643.s013.tif]

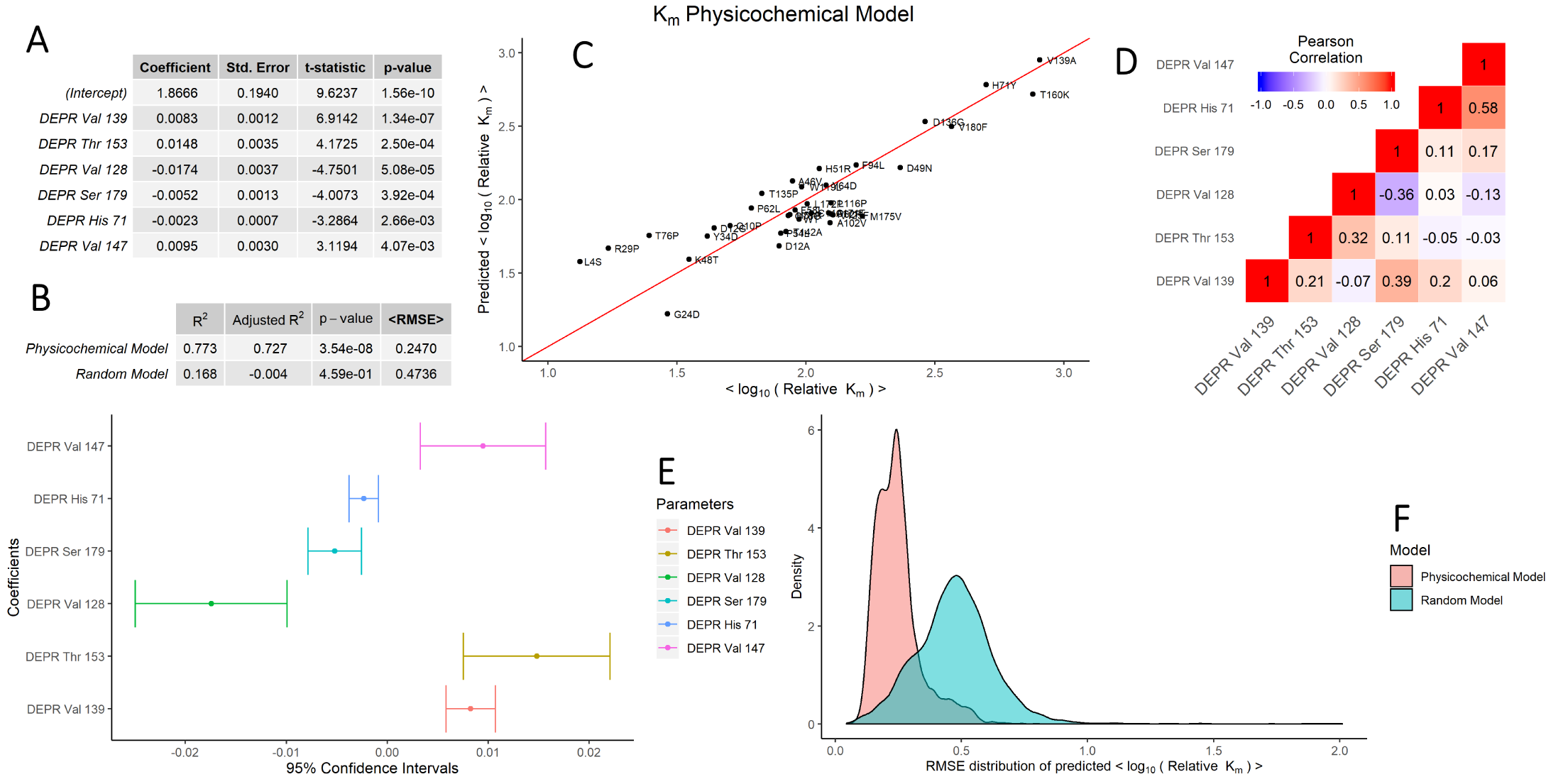

Supplement: S14 Fig — (A) Table with estimated coefficients and statistics for the selected physicochemical descriptors (DEPRs). (B) Comparison of statistics (R2, Adjusted R2, P-value, and RMSE) between the physicochemical model and a random physicochemical model. (C) Fitted values and experimental values for mean log10 (relative-KM). (D) Heatmap showing the correlation coefficient between the selected descriptors. (E) Confidence intervals for the coefficients of each physicochemical descriptor. (F) Distribution of RMSEs calculated by 6-fold cross-validation for the physicochemical model (red) and a random model (blue). (TIF) [file pone.0235643.s014.tif]

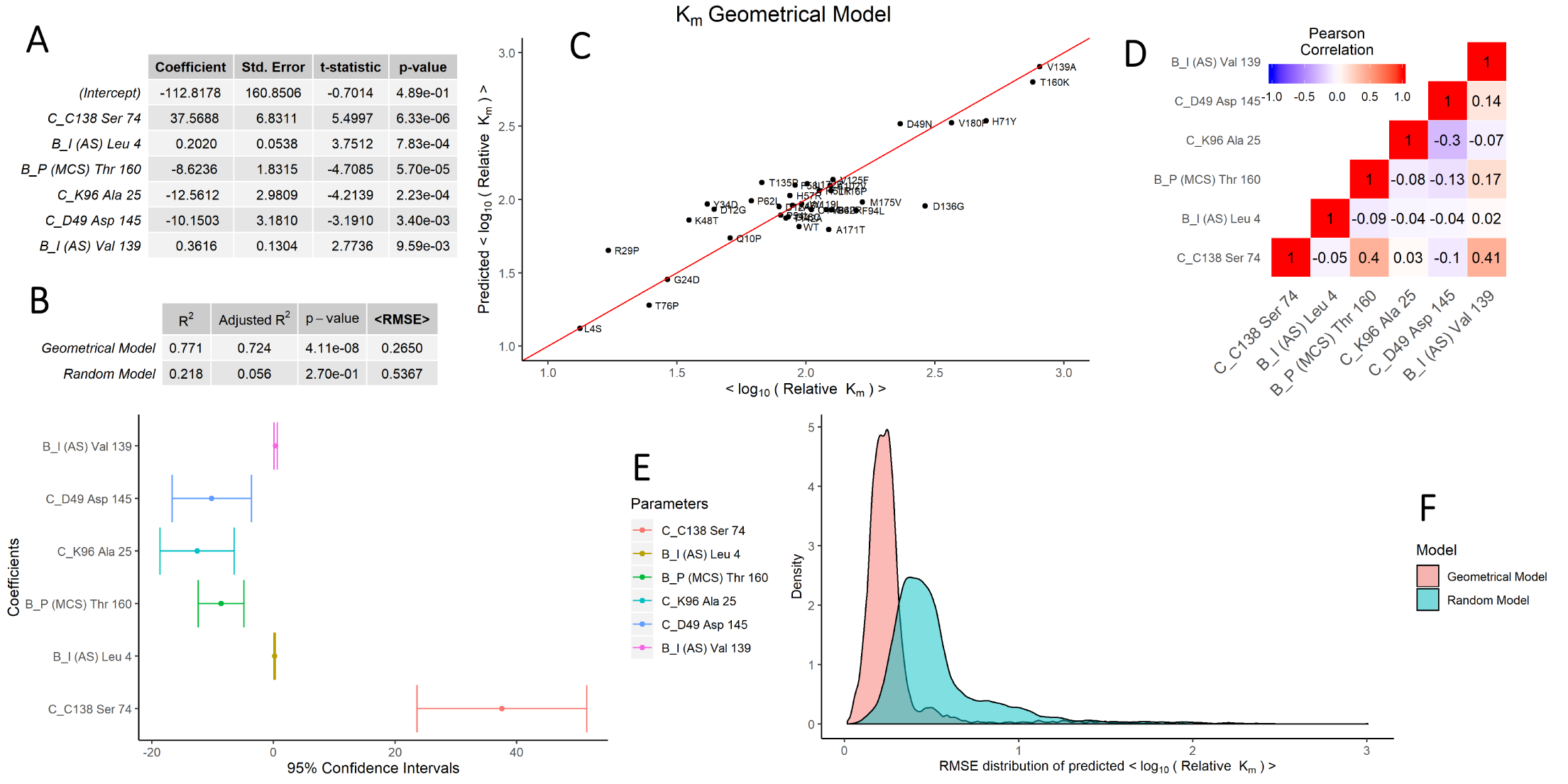

Supplement: S15 Fig — (A) Table with estimated coefficients and statistics for the selected geometrical descriptors. (B) Comparison of statistics (R2, Adjusted R2, P-value, and RMSE) between the geometrical model and a random geometrical model. (C) Fitted values and experimental values for mean log10 (relative-KM). (D) Heatmap showing the correlation coefficient between the selected descriptors. (E) Confidence intervals for the coefficients of each geometrical descriptor. (F) Distribution of RMSEs calculated by 6-fold cross-validation for the geometrical model (red) and a random model (blue). (TIF) [file pone.0235643.s015.tif]

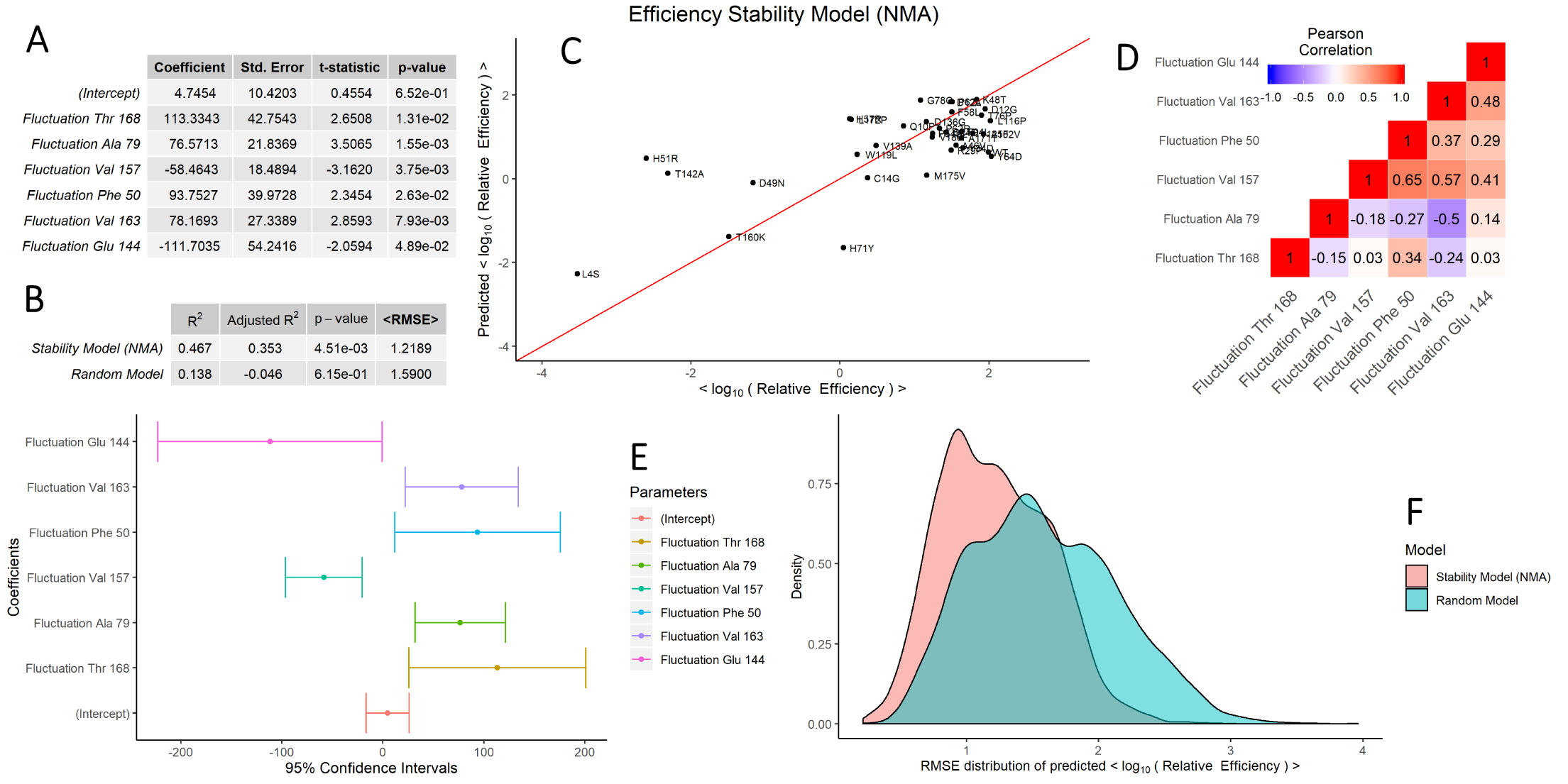

Supplement: S16 Fig — (A) Table with estimated coefficients and statistics for the selected stability descriptors (fluctuations). (B) Comparison of statistics (R2, Adjusted R2, P-value, and RMSE) between the stability model and a random stability model. (C) Fitted values and experimental values for mean log10 (relative efficiency). (D) Heatmap showing the correlation coefficient between the selected descriptors. (E) Confidence intervals for the coefficients of each stability descriptor. (F) Distribution of RMSEs calculated by 6-fold cross-validation for the stability model (red) and a random model (blue). (TIF) [file pone.0235643.s016.tif]

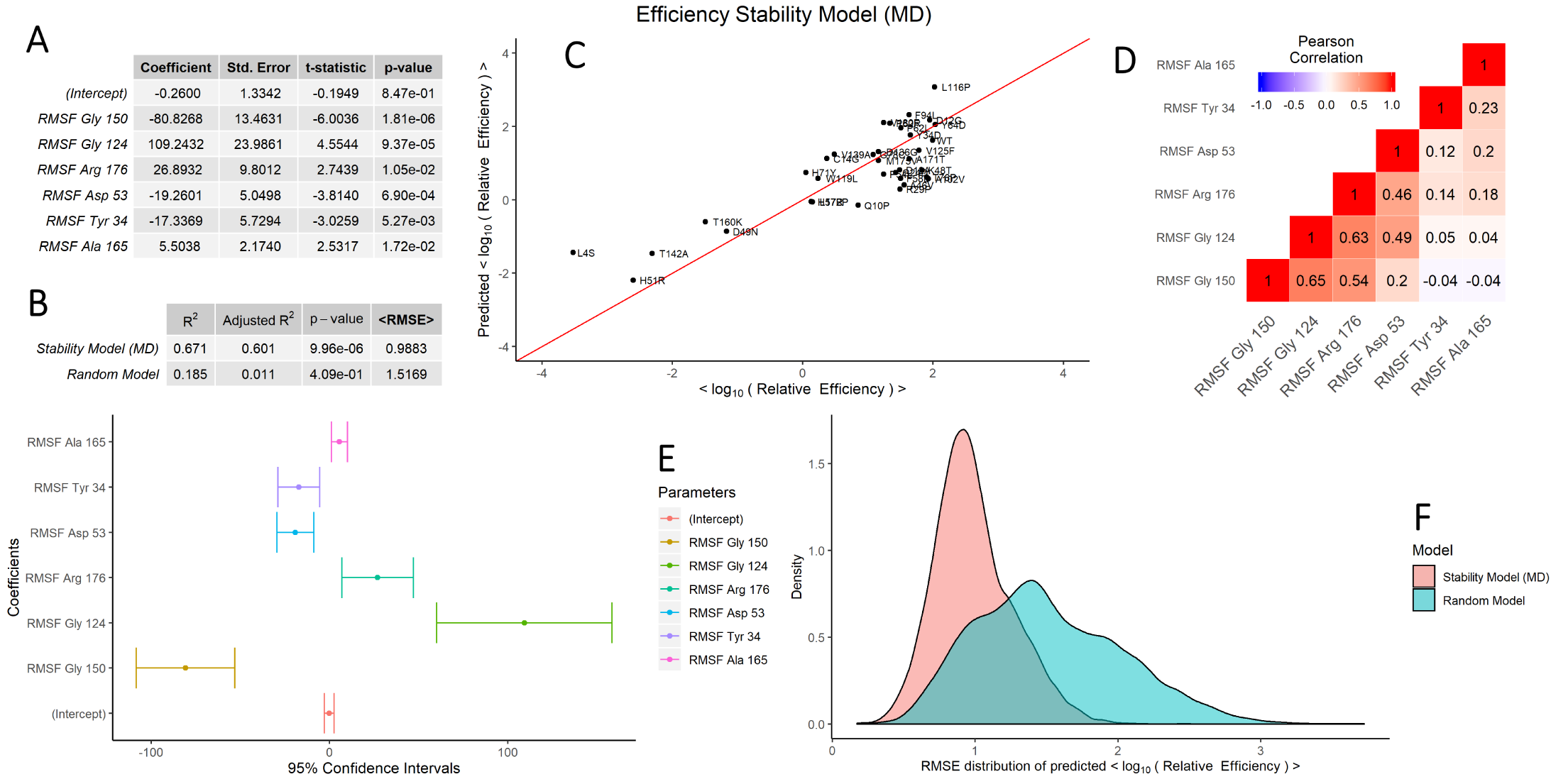

Supplement: S17 Fig — (A) Table with estimated coefficients and statistics for the selected stability descriptors (RMSFs). (B) Comparison of statistics (R2, Adjusted R2, P-value, and RMSE) between the stability model and a random stability model. (C) Fitted values and experimental values for mean log10 (relative efficiency). (D) Heatmap showing the correlation coefficient between the selected descriptors. (E) Confidence intervals for the coefficients of each stability descriptor. (F) Distribution of RMSEs calculated by 6-fold cross-validation for the stability model (red) and a random model (blue). (TIF) [file pone.0235643.s017.tif]

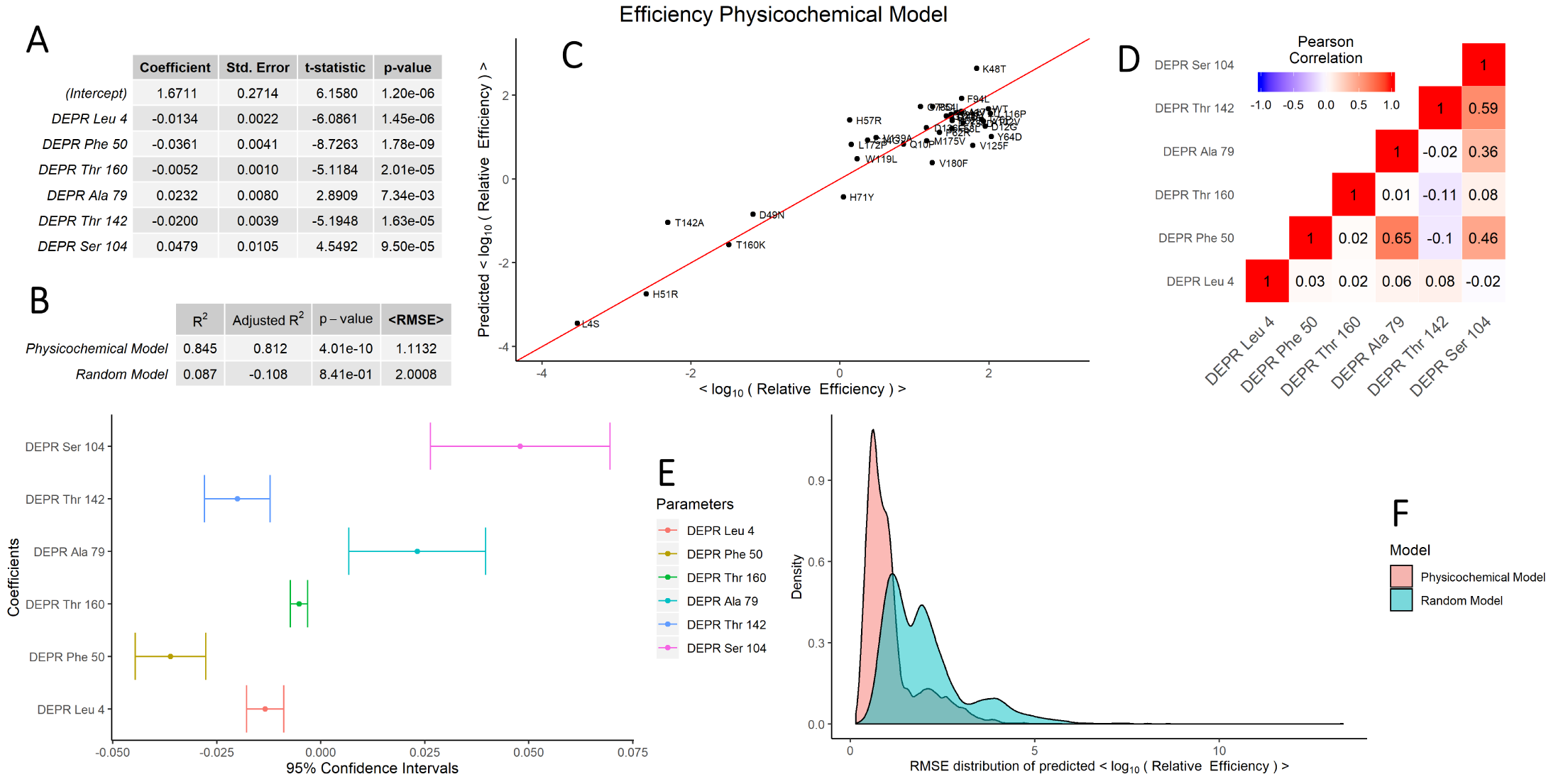

Supplement: S18 Fig — (A) Table with estimated coefficients and statistics for the selected physicochemical descriptors (DEPRs). (B) Comparison of statistics (R2, Adjusted R2, P-value, and RMSE) between the physicochemical model and a random physicochemical model. (C) Fitted values and experimental values for mean log10 (relative efficiency). (D) Heatmap showing the correlation coefficient between the selected descriptors. (E) Confidence intervals for the coefficients of each physicochemical descriptor. (F) Distribution of RMSEs calculated by 6-fold cross-validation for the physicochemical model (red) and a random model (blue). (TIF) [file pone.0235643.s018.tif]

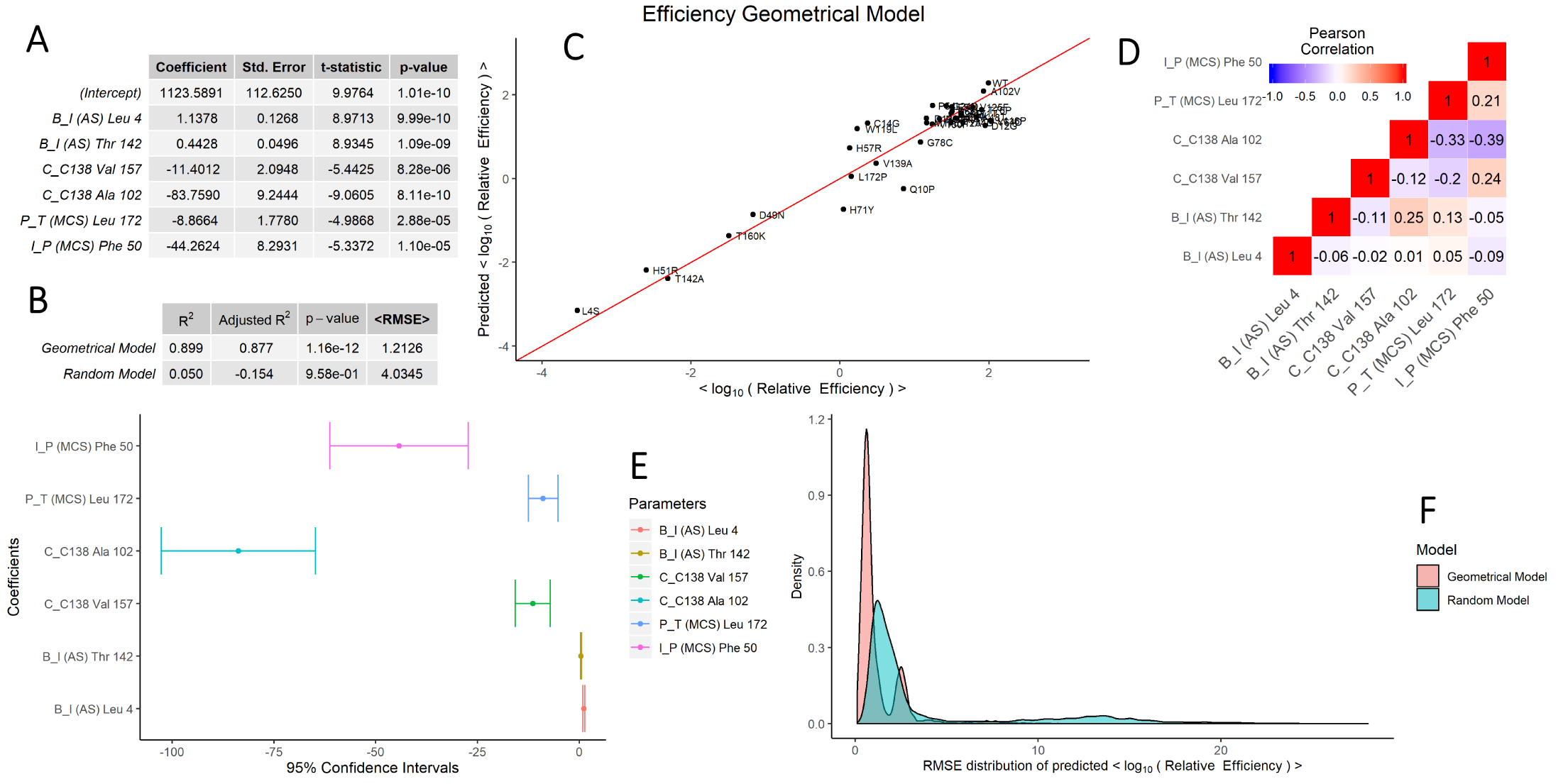

Supplement: S19 Fig — (A) Table with estimated coefficients and statistics for the selected geometrical descriptors. (B) Comparison of statistics (R2, Adjusted R2, P-value, and RMSE) between the geometrical model and a random geometrical model. (C) Fitted values and experimental values for mean log10 (relative efficiency). (D) Heatmap showing the correlation coefficient between the selected descriptors. (E) Confidence intervals for the coefficients of each geometrical descriptor. (F) Distribution of RMSEs calculated by 6-fold cross-validation for the geometrical model (red) and a random model (blue). (TIF) [file pone.0235643.s019.tif]

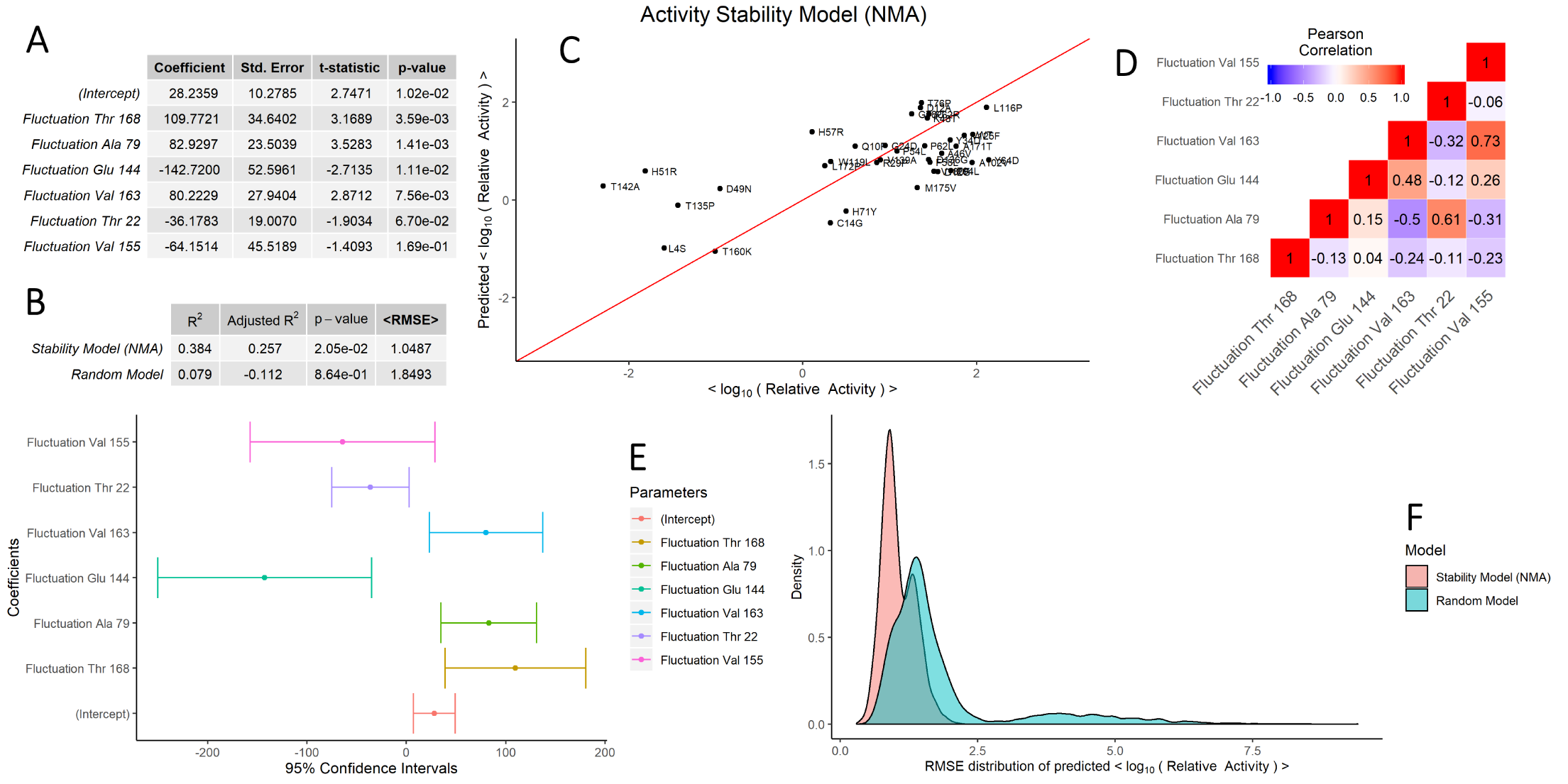

Supplement: S20 Fig — (A) Table with estimated coefficients and statistics for the selected stability descriptors (fluctuations). (B) Comparison of statistics (R2, Adjusted R2, P-value, and RMSE) between the stability model and a random stability model. (C) Fitted values and experimental values for mean log10 (relative activity). (D) Heatmap showing the correlation coefficient between the selected descriptors. (E) Confidence intervals for the coefficients of each stability descriptor. (F) Distribution of RMSEs calculated by 6-fold cross-validation for the stability model (red) and a random model (blue). (TIF) [file pone.0235643.s020.tif]

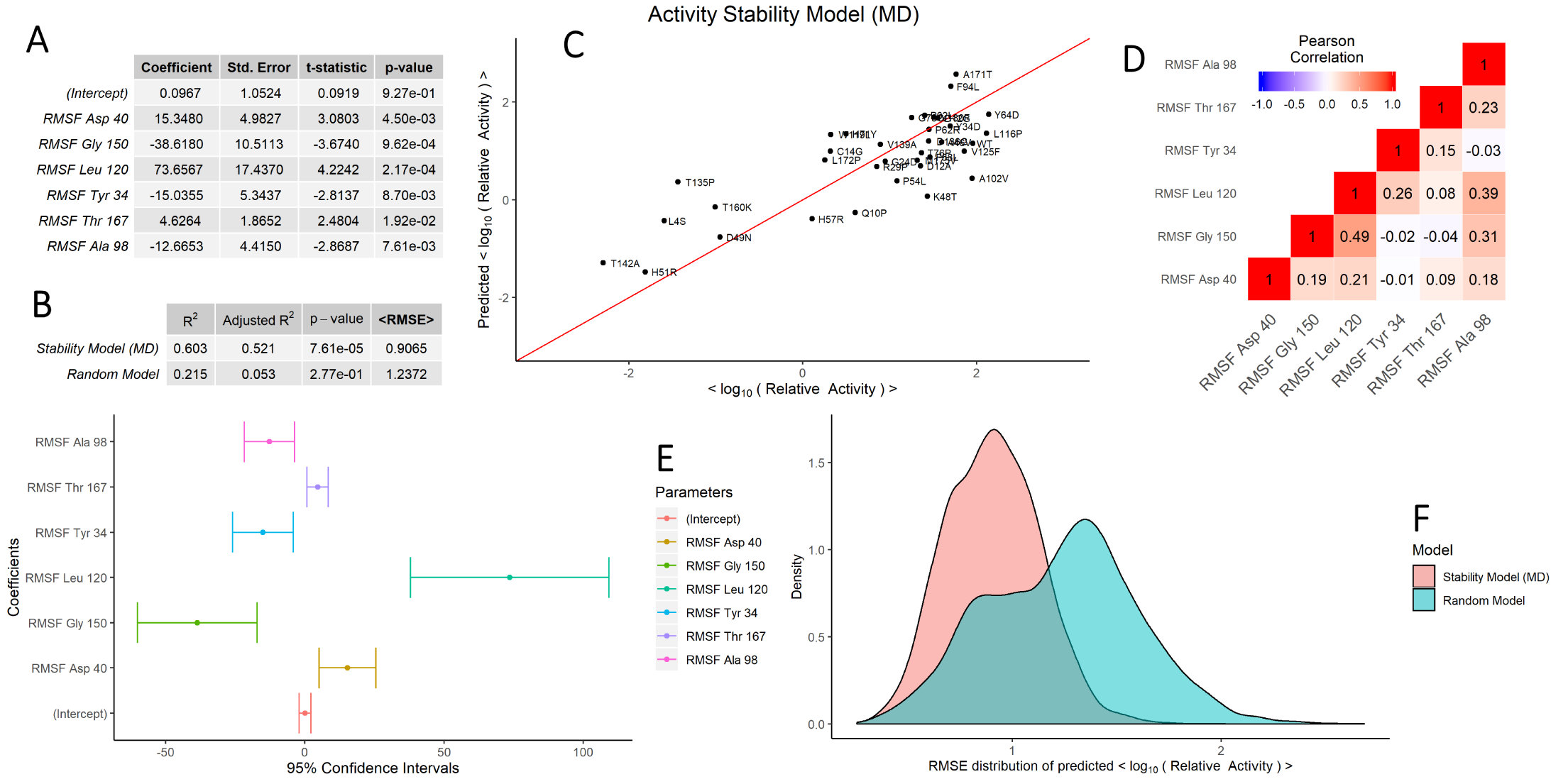

Supplement: S21 Fig — (A) Table with estimated coefficients and statistics for the selected stability descriptors (RMSFs). (B) Comparison of statistics (R2, Adjusted R2, P-value, and RMSE) between the stability model and a random stability model. (C) Fitted values and experimental values for mean log10 (relative activity). (D) Heatmap showing the correlation coefficient between the selected descriptors. (E) Confidence intervals for the coefficients of each stability descriptor. (F) Distribution of RMSEs calculated by 6-fold cross-validation for the stability model (red) and a random model (blue). (TIF) [file pone.0235643.s021.tif]

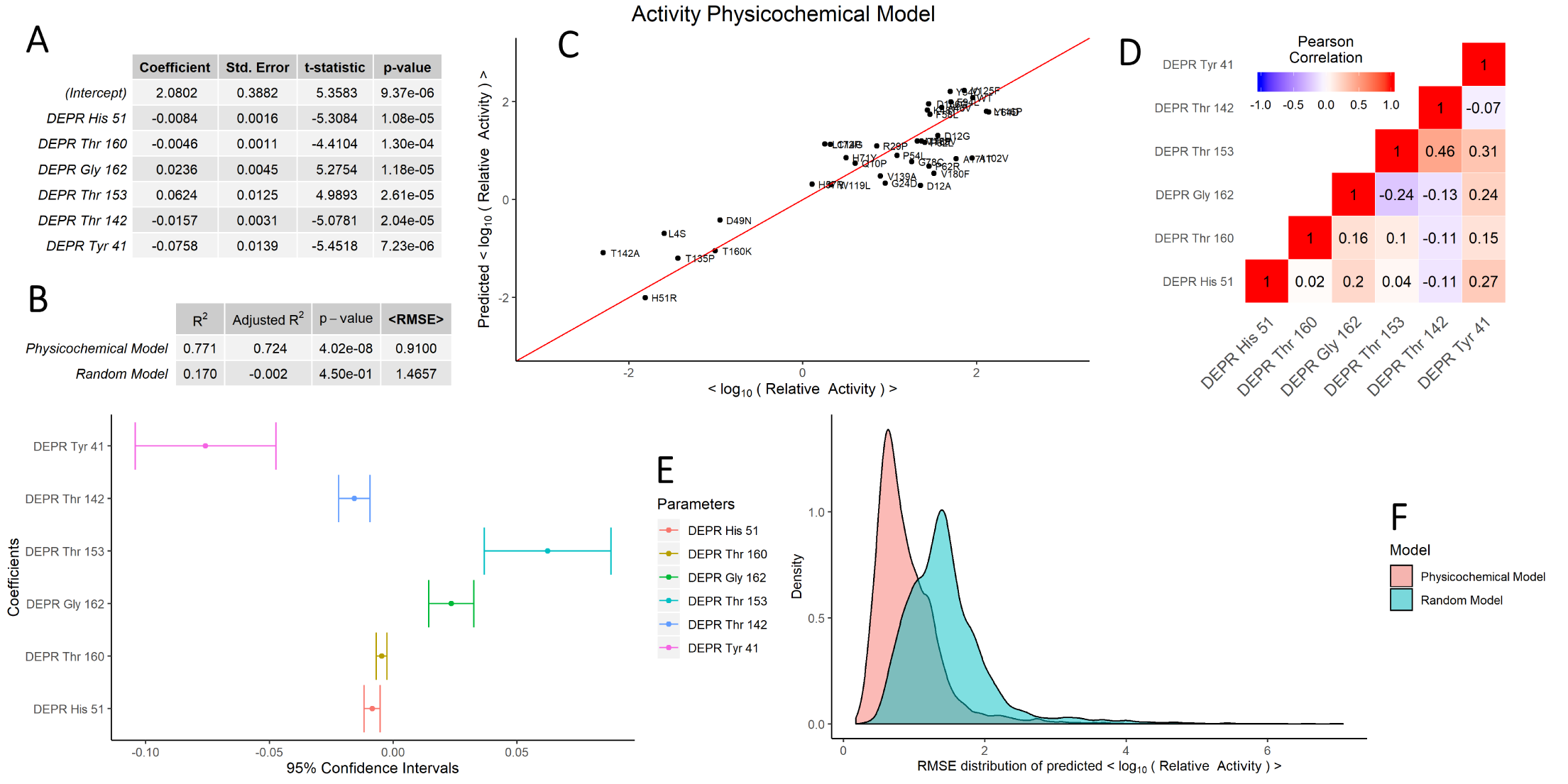

Supplement: S22 Fig — (A) Table with estimated coefficients and statistics for the selected physicochemical descriptors (DEPRs). (B) Comparison of statistics (R2, Adjusted R2, P-value, and RMSE) between the physicochemical model and a random physicochemical model. (C) Fitted values and experimental values for mean log10 (relative activity). (D) Heatmap showing the correlation coefficient between the selected descriptors. (E) Confidence intervals for the coefficients of each physicochemical descriptor. (F) Distribution of RMSEs calculated by 6-fold cross-validation for the physicochemical model (red) and a random model (blue). (TIF) [file pone.0235643.s022.tif]

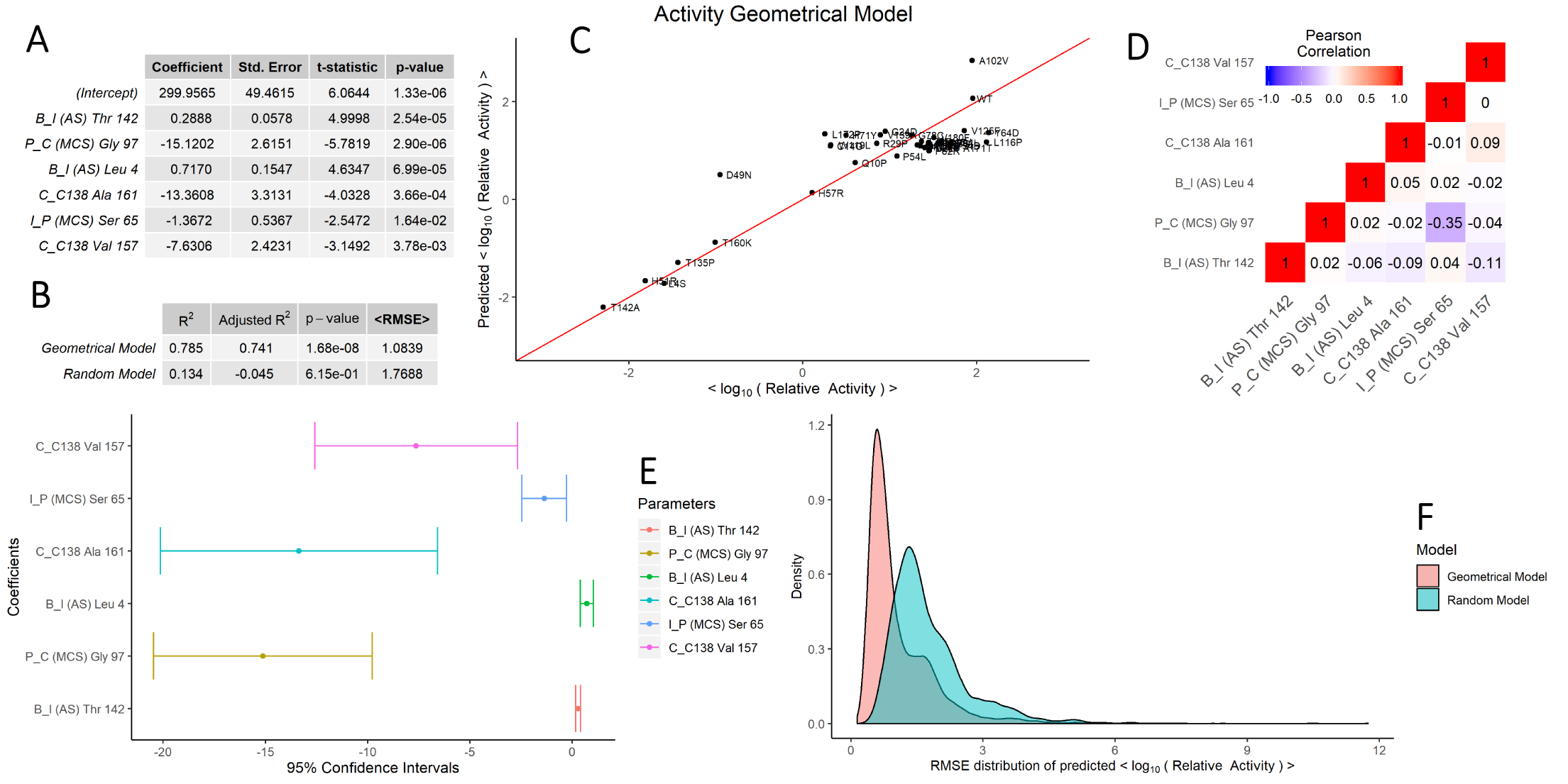

Supplement: S23 Fig — (A) Table with estimated coefficients and statistics for the selected geometrical descriptors. (B) Comparison of statistics (R2, Adjusted R2, P-value00, and RMSE) between the geometrical model and a random geometrical model. (C) Fitted values and experimental values for mean log10 (relative activity). (D) Heatmap showing the correlation coefficient between the selected descriptors. (E) Confidence intervals for the coefficients of each geometrical descriptor. (F) Distribution of RMSEs calculated by 6-fold cross-validation for the geometrical model (red) and a random model (blue). (TIF) [file pone.0235643.s023.tif]

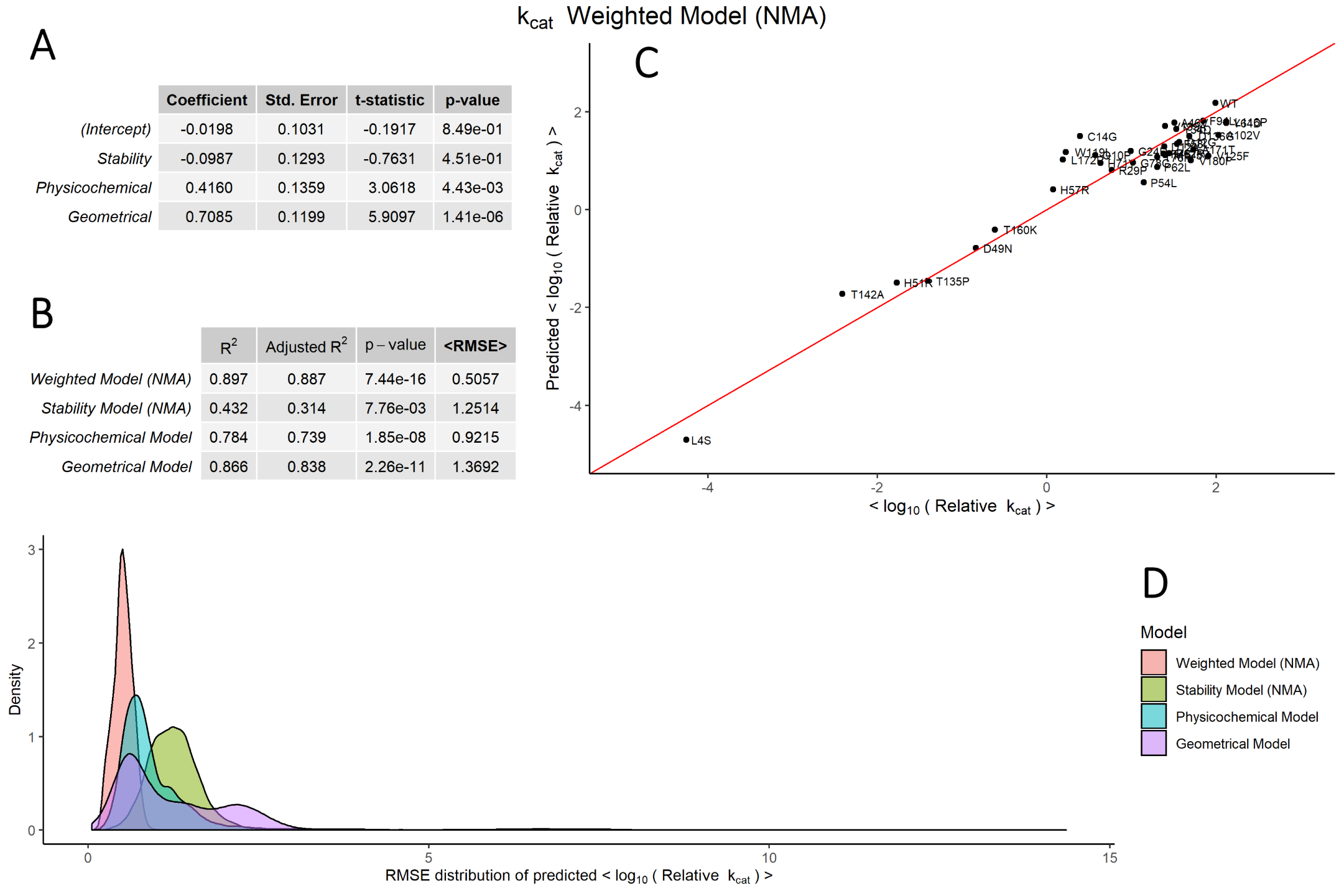

Supplement: S24 Fig — (A) Table with estimated coefficients and statistics for the individual predictions of stability (NMA), physicochemical and geometrical models. (B) Comparison among the individual models and the weighted model (NMA) for kcat. (C) Fitted values and experimental values for mean log10 (relative-kcat) for the weighted model (NMA). (D) Distribution of RMSE calculated by 6-fold cross-validation for the weighted and individual models. (TIF) [file pone.0235643.s024.tif]

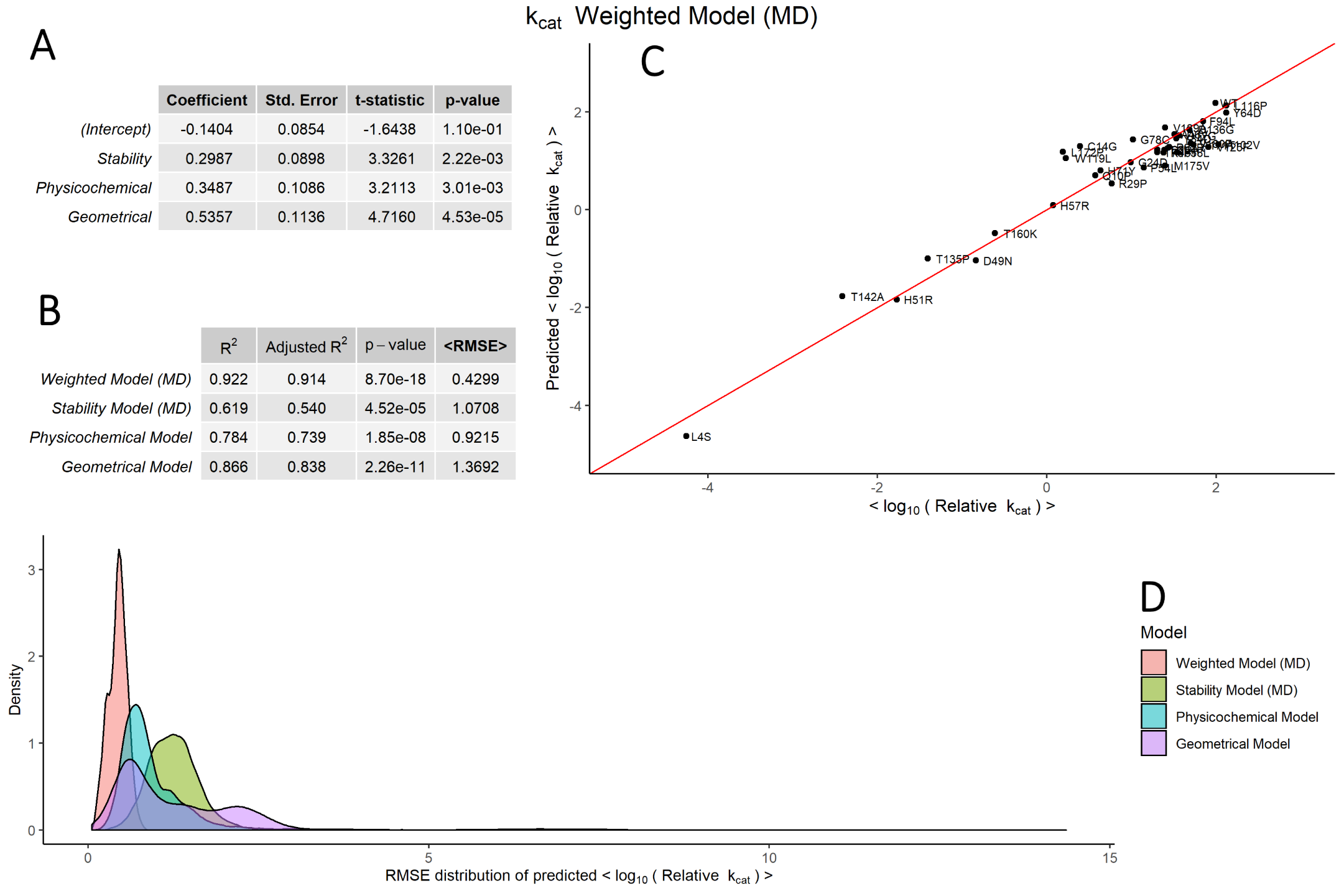

Supplement: S25 Fig — (A) Table with estimated coefficients and statistics for the individual predictions of stability (MD), physicochemical and geometrical models. (B) Comparison among the individual models and the weighted model (MD) for kcat. (C) Fitted values and experimental values for mean log10 (relative-kcat) for the weighted model (MD). (D) Distribution of RMSE calculated by 6-fold cross-validation for the weighted and individual models. (TIF) [file pone.0235643.s025.tif]

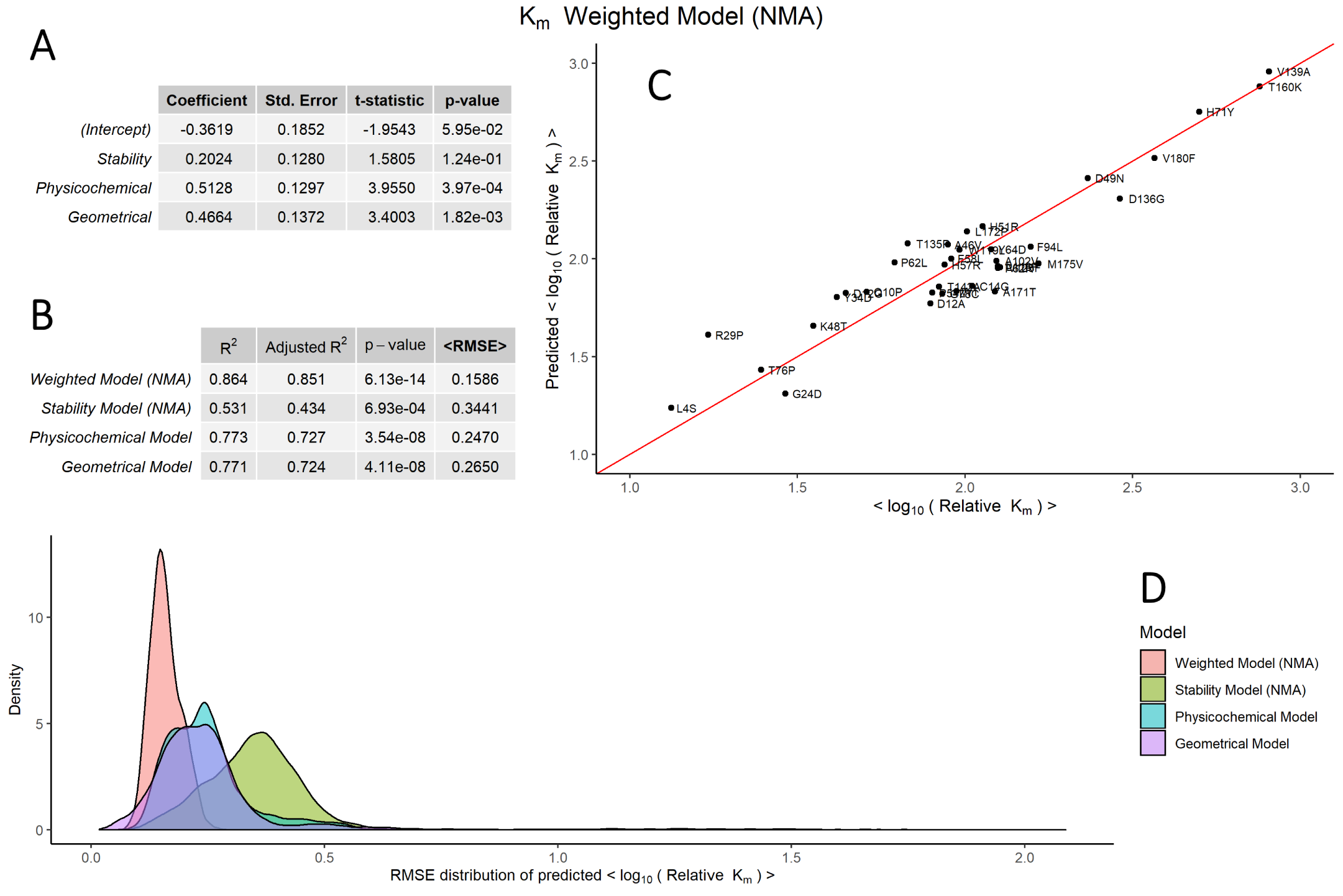

Supplement: S26 Fig — (A) Table with estimated coefficients and statistics for the individual predictions of stability (NMA), physicochemical and geometrical models. (B) Comparison among the individual models and the weighted model (NMA) for KM. (C) Fitted values and experimental values for mean log10 (relative- KM) for the weighted model (NMA). (D) Distribution of RMSE calculated by 6-fold cross-validation for the weighted and individual models. (TIF) [file pone.0235643.s026.tif]

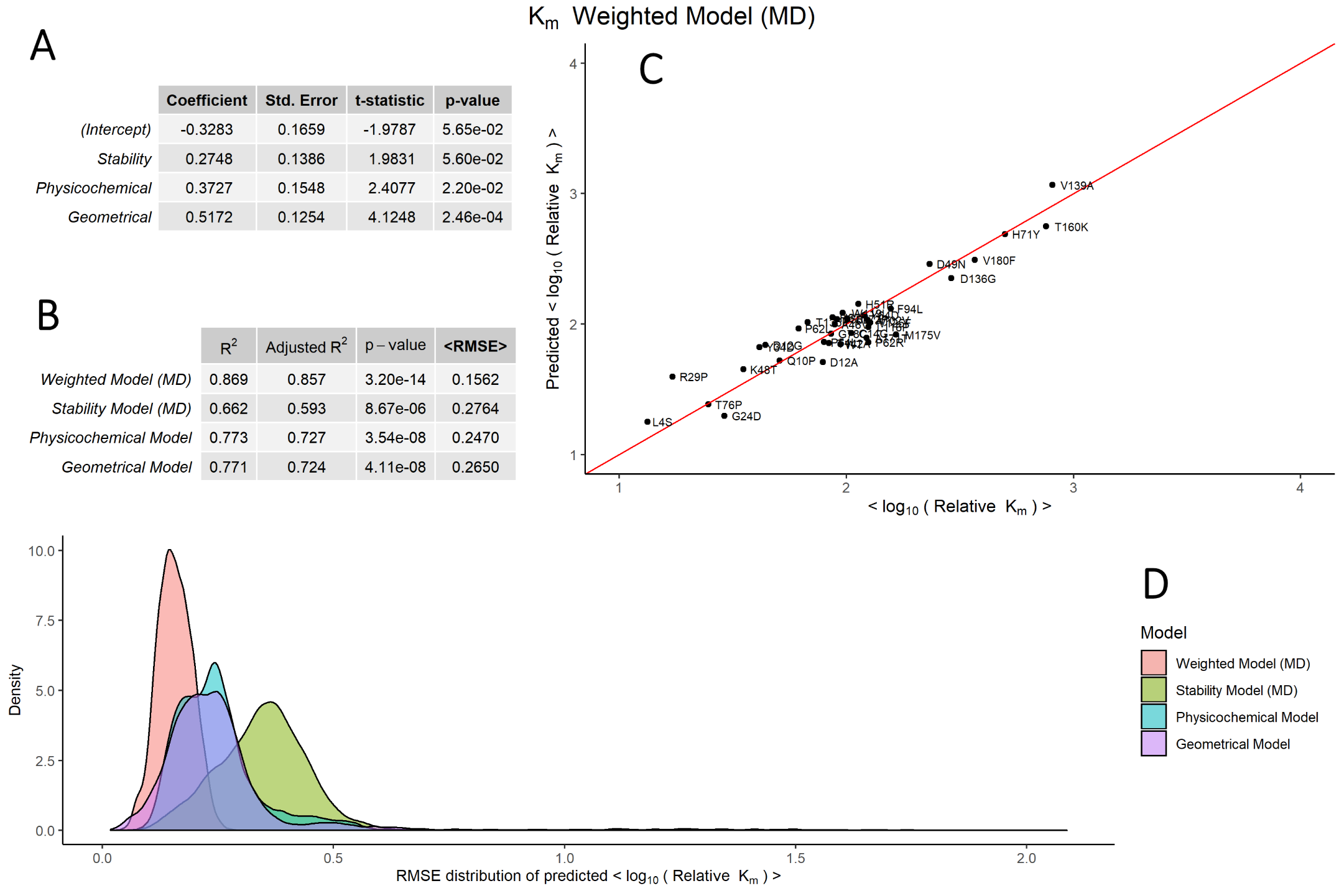

Supplement: S27 Fig — (A) Table with estimated coefficients and statistics for the individual predictions of stability (MD), physicochemical and geometrical models. (B) Comparison among the individual models and the weighted model (MD) for KM. (C) Fitted values and experimental values for mean log10 (relative-KM) for the weighted model (MD). (D) Distribution of RMSE calculated by 6-fold cross-validation for the weighted and individual models. (TIF) [file pone.0235643.s027.tif]

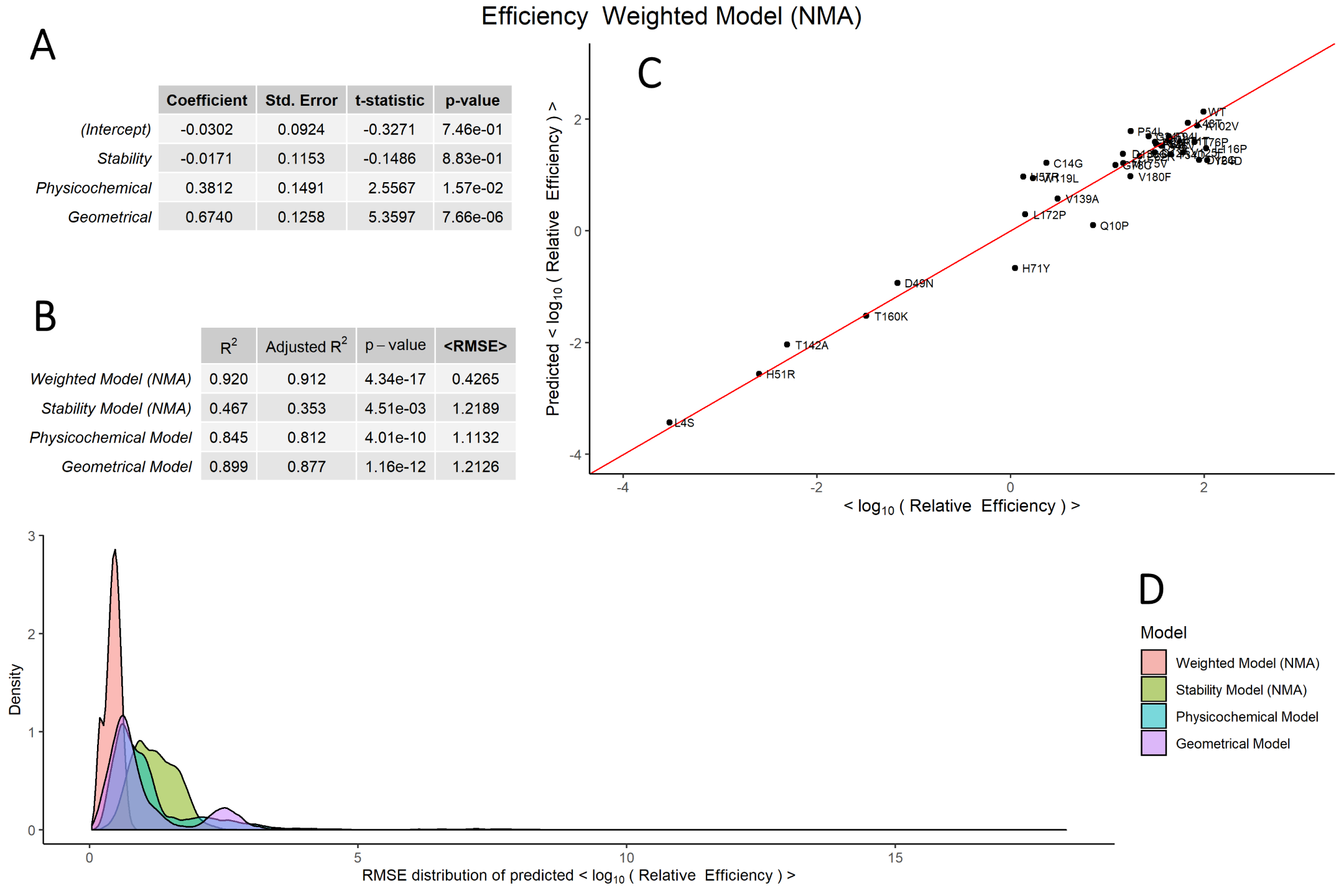

Supplement: S28 Fig — (A) Table with estimated coefficients and statistics for the individual predictions of stability (NMA), physicochemical and geometrical models. (B) Comparison among the individual models and the weighted model (NMA) for efficiency. (C) Fitted values and experimental values for mean log10 (relative efficiency) for the weighted model (NMA). (D) Distribution of RMSE calculated by 6-fold cross-validation for the weighted and individual models. (TIF) [file pone.0235643.s028.tif]

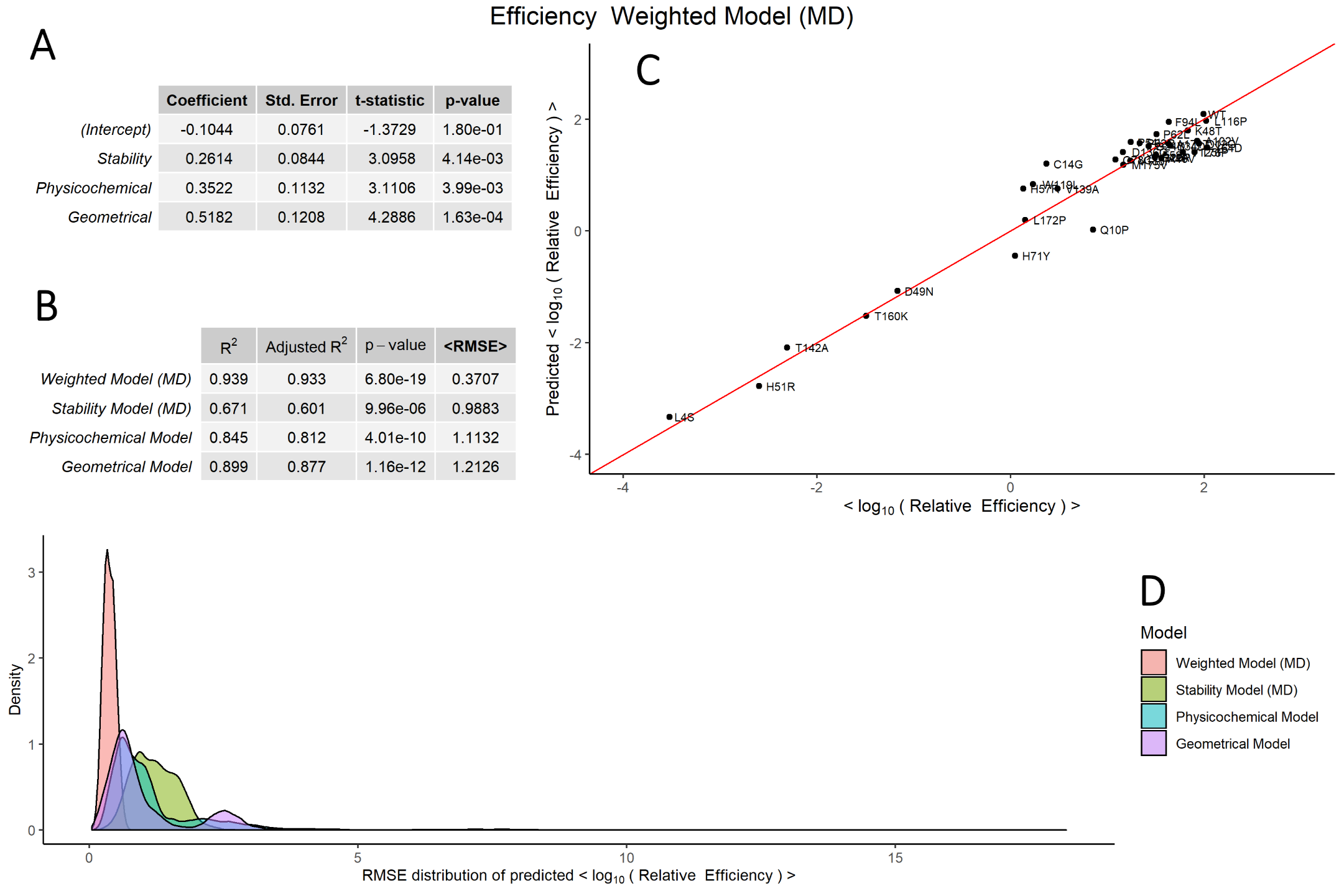

Supplement: S29 Fig — (A) Table with estimated coefficients and statistics for the individual predictions of stability (MD), physicochemical and geometrical models. (B) Comparison among the individual models and the weighted model (MD) for efficiency. (C) Fitted values and experimental values for mean log10 (relative efficiency) for the weighted model (MD). (D) Distribution of RMSE calculated by 6-fold cross-validation for the weighted and individual models. (TIF) [file pone.0235643.s029.tif]

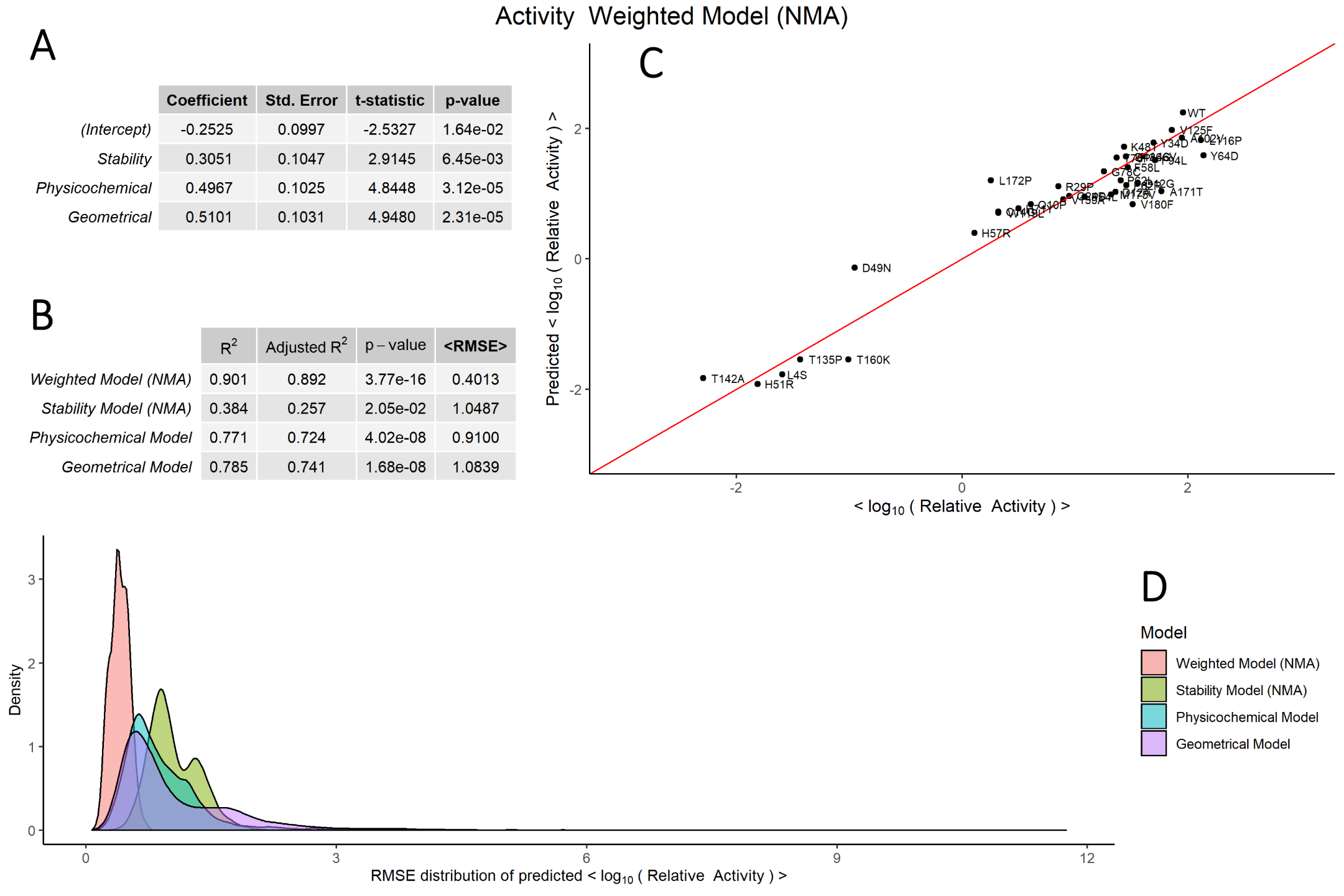

Supplement: S30 Fig — (A) Table with estimated coefficients and statistics for the individual predictions of stability (NMA), physicochemical and geometrical models. (B) Comparison among the individual models and the weighted model (NMA) for activity. (C) Fitted values and experimental values for mean log10 (relative activity) for the weighted model (NMA). (D) Distribution of RMSE calculated by 6-fold cross-validation for the weighted and individual models. (TIF) [file pone.0235643.s030.tif]

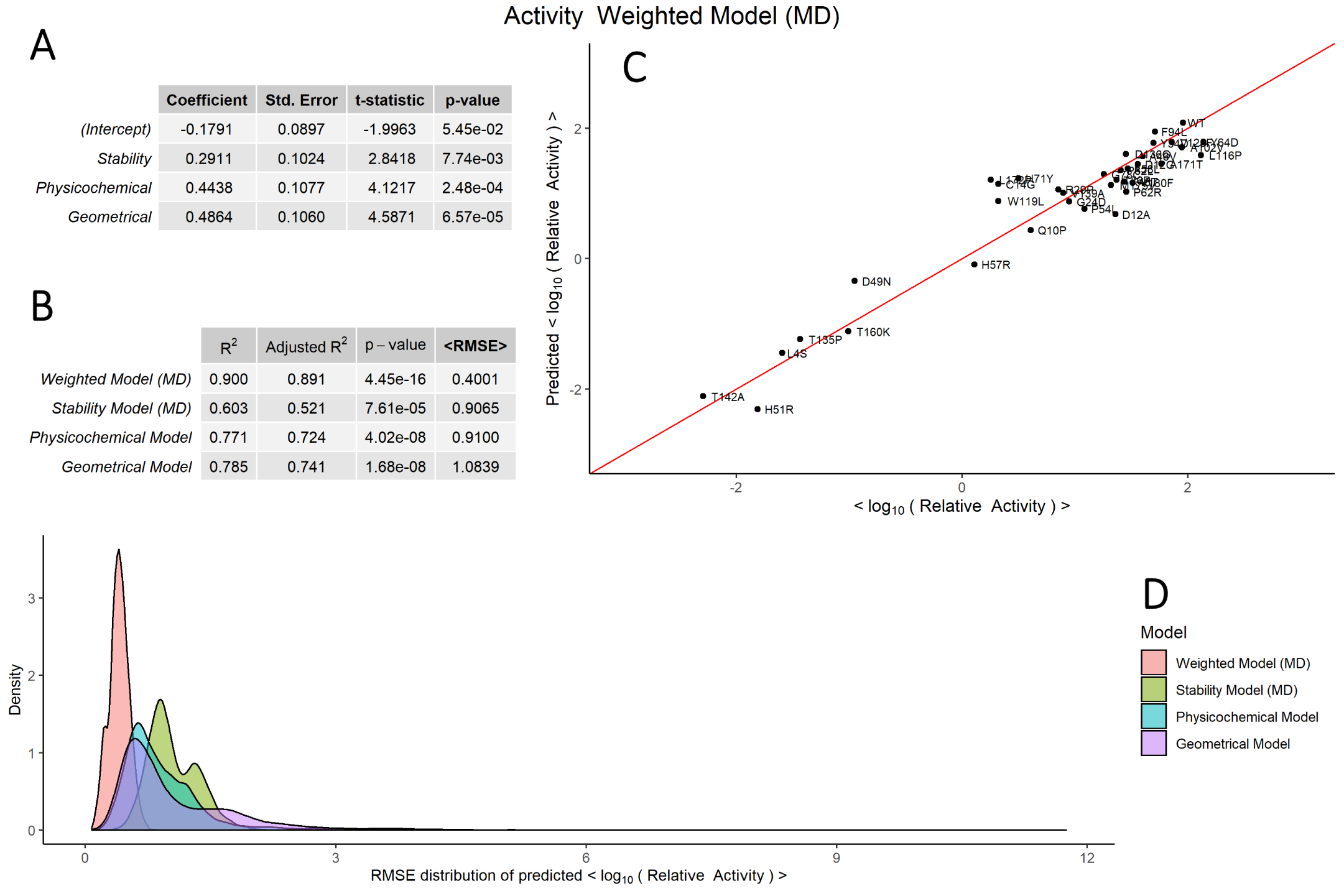

Supplement: S31 Fig — (A) Table with estimated coefficients and statistics for the individual predictions of stability (MD), physicochemical and geometrical models. (B) Comparison among the individual models and the weighted model (MD) for activity. (C) Fitted values and experimental values for mean log10 (relative activity) for the weighted model (MD). (D) Distribution of RMSE calculated by 6-fold cross-validation for the weighted and individual models. (TIF) [file pone.0235643.s031.tif]
